# Vancomycin and/or Multidrug-Resistant *Citrobacter Freundii* Altered the Metabolic Pattern of Soil Microbial Community

**DOI:** 10.3389/fmicb.2018.01047

**Published:** 2018-05-23

**Authors:** Mariusz Cycoń, Kamila Orlewska, Anna Markowicz, Agnieszka Żmijowska, Joanna Smoleń-Dzirba, Jolanta Bratosiewicz-Wąsik, Tomasz J. Wąsik, Zofia Piotrowska-Seget

**Affiliations:** ^1^Department of Microbiology and Virology, School of Pharmacy with the Division of Laboratory Medicine, Medical University of Silesia, Sosnowiec, Poland; ^2^Department of Microbiology, University of Silesia, Katowice, Poland; ^3^Department of Ecotoxicology, Institute of Industrial Organic Chemistry, Pszczyna, Poland

**Keywords:** vancomycin, multidrug-resistant bacteria, Biolog EcoPlates, enzyme activities, antibiotic dissipation, soil

## Abstract

Despite many studies, our knowledge on the impact of antibiotics and antibiotic-resistant bacteria on the metabolic activity of soil microbial communities is still limited. To ascertain this impact, the community level physiological profiles (CLPPs) and the activity of selected enzymes (dehydrogenase, urease, and phosphatases) in soils treated with vancomycin (VA) and/or multidrug resistant *Citrobacter freundii* were determined during a 90-day experiment. A multivariate analysis and the resistance (RS)/resilience (RL) concept were used to assess the potential of native microorganisms to maintain their catabolic activity under exposure of VA and/or a high level of *C. freundii*. In addition, the dissipation rate of VA was evaluated in non-sterile (nsS) and sterile (sS) soils. The results revealed a negative impact of VA on the metabolic activity of soil microorganisms on days 1, 15, and 30 as was showed by a decrease in the values of the CLPP indices (10–69%) and the enzyme activities (6–32%) for treated soils as compared to the control. These observations suggested a low initial resistance of soil microorganisms to VA and/or *C. freundii* but they were resilient in the long term. Considering the mean values of the RS index, the resistance of measured parameters was categorized in the following order: alkaline phosphatase (0.919) > acid phosphatase (0.899) > dehydrogenase (0.853) > the evenness index (0.840) > urease (0.833) > the Shannon-Wiener index (0.735) > substrate richness (0.485) > the AWCD (0.301). The dissipation process of VA was relatively fast and independent of the concentration used. The DT50 values for VA applied at both concentrations were about 16 days. In addition, the dissipation of VA in nsS was three times faster compared to the dissipation of antibiotic in sS. In conclusion, both CLPP and enzyme activities assays appeared to be useful tool for the determination of disturbances within soil microbial communities and used together may be helpful to understand the changes in their catabolic features. The entry of large quantities of VA and/or *C*. *freundii* into soil may temporarily change microbial activity thus pose a potential risk for soil functioning.

## Introduction

Antibiotics and antibiotic-resistant microorganisms are primarily introduced into soil through the manure, municipal wastewater, or sewage sludge application (Kümmerer, 2003; Chee-Sanford et al., 2009; Li and Zhang, 2010). Moreover, several antibiotics are used and overused in agriculture practices (Chang and Ren, 2015). Due to their potential ecotoxicological effects and persistence in soil, antibiotics represent a durable contamination (Brandt et al., 2015). The presence of antibiotics in soil poses a prospective risk to ecosystem health because of the selective pressure that is exerted on soil microbial communities. Many studies have revealed that antibiotics affect the number of different groups of microorganisms (Pinna et al., 2012; Akimenko et al., 2015; Xu et al., 2016), the structural and genetic diversity of microorganisms and the overall microbial activity (Demoling et al., 2009; Cui et al., 2013; Liu et al., 2014; Reichel et al., 2014a,b; Cycoń et al., 2016a; Xu et al., 2016). Moreover, the impact of antibiotics on the enzyme activities, carbon mineralization, and nitrogen cycling has been proven (Liu et al., 2009; Kotzerke et al., 2011; Rosendahl et al., 2012; Chen et al., 2013; Ma et al., 2016).

The potential disturbances/alterations within soil microorganisms caused by various stress factors may be assessed using the resistance (RS)/resilience (RL) concept (Orwin and Wardle, 2004; Griffiths and Philippot, 2013). Resistance means the ability of a microbial community to maintain the population structure and function under a toxicity stress, whereas resilience is defined as the ability of community to recover from a perturbation or disturbance to its original or new stable composition and functionality (Allison and Martiny, 2008; Shade et al., 2012; Hodgson et al., 2015; Song et al., 2015). Soil microorganisms are faced with both abiotic (e.g., pollutants, physico-chemical factors) and biotic (e.g., bacteriophages, competition with other organisms) stressors. Although multiple stress factors typically co-occur in soil system, studies on their impact on microbial communities are limited. It has been reported that the resilience of polluted soils against further stress is different than those observed in non-contaminated soils (Schaeffer et al., 2016). An understanding of the reaction of soil microbial communities to biotic and abiotic stressors acting simultaneously is currently lacking.

Vancomycin (VA) is a glycopeptide antibiotic that inhibits the cell wall synthesis by affecting the peptidoglycan assembly. This mechanism leads to the inhibition of bacterial cell division (Courvalin, 2006; Gupta et al., 2011). VA has increasingly been used against different infections caused by Gram-positive bacteria in recent decades and has been detected in hospital effluents worldwide (Qiu et al., 2016; Quoc Tuc et al., 2017). Since conventional wastewater treatment processes have a limited efficiency in VA dissipation, this antibiotic may enter the environment via the final release of effluents and the application of sewage sludge into soil (Quoc Tuc et al., 2017). Moreover, multidrug-resistant strains in soil may have the same origin. In consequence, the presence of VA or other antibiotics in soil may favor the growth and spread of resistant microorganisms. Multidrug-resistant strains of species such as *Citrobacter freundii* that are a part of the indigenous soil microbial communities have an advantage (Riber et al., 2014). Because *C. freundii* is also known to be an opportunistic pathogen, it is important to understand the impact of VA and/or multidrug-resistant *C. freundii* on the functional and structural diversity of natural soil microorganisms. Based on the denaturing gradient gel electrophoresis (DGGE) and phospholipid fatty acid (PLFA) approaches, we revealed that VA and/or multidrug-resistant *C. freundii* changed the structure and genetic biodiversity of a soil microbial community (Cycoń et al., 2016a).

Since the basic soil functions such as biomass production and nutrient turnover, biogeochemical cycling and soil formation are mainly provided by microorganisms, it is important to learn the resistance/resilience of soil activity to application of the antibiotic and a high number of antibiotic-resistant bacteria. In order to obtain knowledge about the metabolic potential of soil microorganisms, referred to as the community-level physiological profile (CLPP), the Biolog method and EcoPlates™ that contain 31 various carbon sources can be used (Garland, 1997; Floch et al., 2011). The CLPP approach has often been used to determine the microbial catabolic activity and functional diversity in soil that has been contaminated with different antibiotics and other chemicals (Mijangos et al., 2009; Liu W. et al., 2012; Cycoń et al., 2013a; Chessa et al., 2016; Fang et al., 2016). However, some authors stated that the effects of contamination can be better evaluated by measuring the activity of some soil enzymes rather than use of Biolog EcoPlates (Floch et al., 2011; Cycoń et al., 2013b). This conclusion may be due to the fact that the Biolog technique does not take into account the activity of catabolically inactive microorganisms that exist in a dormant state or non-culturable microorganisms. Moreover, mainly fast growing microorganisms are involved in this analysis (Floch et al., 2011). Many studies have indicated that dehydrogenase, phosphatase and urease activities are sensitive indicators of the microbial response to stress that is caused by antibiotics in the soil environment (Yang et al., 2009; Akimenko et al., 2015; Xu et al., 2016). However, interactions between the antibiotic and/or multidrug-resistant strain and the soil microbial biochemical potential are still little known. In this context, the application of VA and/or antibiotic-resistant bacteria into soil may change the biochemical potential of a soil microbial community. In this study we hypothesized that the introduction of VA and/or multidrug-resistant *C. freundii* into soil could shift the metabolic activity of a microbial community and the presence of a high number of antibiotic-resistant bacteria could change the response of indigenous microrganisms to VA. To check the above assumptions, the CLPP and enzyme activities, i.e., dehydrogenase (DHA), acid phosphatase (PHOS-H), alkaline phosphatase (PHOS-OH), and urease (URE) were determined. A multivariate analysis and the resistance (RS)/resilience (RL) concept were used to assess the potential of native microorganisms to maintain their catabolic activity under exposure of VA and/or a high level of *C. freundii*. In addition, an analysis of the VA dissipation in soil was also performed.

## Materials and methods

### Bacterial strain

A raw sewage was used to isolate a bacterial strain. Growth of isolate was performed using a TSA medium in the presence of paper discs impregnated with VA. It was identified as *C. freundii* using the API 20E biochemical test (bioMérieux Inc., France) and 16S rRNA gene analysis with the universal primer pair 27f and 1492r. The *C*. *freundii* strain expressed a resistance to vancomycin, clindamycin and erythromycin. Isolated strain has been deposited in the culture collection of the Department of Microbiology and Virology, Medical University of Silesia, Poland. The standard biosecurity and institutional safety procedures for this bacterial strain have been carried out. Detailed information related to procedures of bacteria isolation and identification were described in a previous paper (Cycoń et al., 2016a).

### Design of experiment

A loamy sand soil was used in the experiment. The properties of the soil were shown in a previous paper (Cycoń et al., 2016a) and determined according to suitable methods (Cycoń et al., 2010). The experiment with the non-sterile soil (nsS) had three replications of each treatment, i.e., C (non-sterile control), VA1 (nsS + 1 mg VA/kg soil), VA10 (nsS + 10 mg VA/kg soil), Cit (nsS + *C*. *freundii*), VA1+Cit (nsS + 1 mg VA/kg soil + *C*. *freundii*), and VA10+Cit (nsS + 10 mg VA/kg soil *C*. *freundii*) for each sampling time. A suspension of a bacterial strain was introduced into the soil treatments at a concentration of 1.6 × 10^7^ cells/g soil (Cycoń et al., 2016a). Soil samples were stored at the temperature of 22 ± 1°C and periodically removed from the test system to evaluate the metabolic pattern and physiological diversity of a bacterial community (on days 1, 15, 30, 60, and 90) and the concentration of VA (on days 0, 1, 8, 15, 23, 30, 60, and 90). Sterile soil (sS) for VA or VA with *C*. *freundii* was used to determine the dissipation of VA under abiotic conditions and the degradation potential of the bacterial strain, respectively. The same experimental conditions were used for both sterile and non-sterile soils. Detailed information related to the design of experiment are presented in Supplementary Materials.

### Analysis of the community-level physiological profile (CLPP)

The CLPP in the soil samples were obtained using the Biolog® EcoPlate™ system (Biolog Inc., CA, USA) (Insam, 1997) and the method described in a previous paper (Cycoń et al., 2013b). Detailed information related to the determination of the CLPP are presented in Supplementary Materials.

### Determination of enzyme activities

The activities of DHA, PHOSs, and URE were determined by methods of Alef (1995), Tabatabai and Bremner (1969), and Gianfreda et al. (1994), respectively, and were described in a previous paper (Cycoń et al., 2016b). Detailed information related to the determination of the enzyme activities are presented in Supplementary Materials.

### Determination of the vancomycin concentration in soil

In order to determine the VA concentration, 10 g soil samples were extracted with 10 mL of a mixture of deionized water/methanol/formic acid (90/10/0.1, v/v) for 5 min. and sonicated for 10 min. Samples were centrifuged and filtered through filter paper. Next, 5 mL of the extraction mixture were added to the soil once again. The procedure of shaking, sonication, centrifugation and filtration was repeated. Finally, 20 μL of the combined extracts were introduced into a chromatographic column. The concentration of VA was determined by high performance liquid chromatography (HPLC) using a Shimadzu Prominence-*i* System LC-2030C 3D (Shimadzu, Inc., Japan) equipped with a DAD detector and a column [Kinetex C18 100A (150 × 4.6 × 5 μm)]. A mixture of acetonitrile/0.05% ortho-phosphoric acid (10/90 v/v) was used as a mobile phase. The detection of VA was performed at a wavelength of 221 nm. The obtained data were analyzed using LabSolution Software LC-2030C 3D. The mean time of retention for VA was 6.1 min.

### Analysis and interpretation of results

Metabolic pattern of a soil microbial community expressed as the average well-color development (AWCD), substrate richness (R_S_), evenness (E), and the Shannon-Wiener index (H) was determined according to the equations described by Garland (1997). Indices adopted from Orwin and Wardle (2004) were used to evaluate the resistance (RS) and resilience (RL) of measured activities to disturbances caused by antibiotic and/or bacterial strain. Based on the analysis of the kinetics of VA dissipation in soil, its disappearance rate was fitted to a zero-order kinetic model. The DT50 values and rate constant (*k*) were calculated by the equation adopted from Cycoń et al. (2013b).

The obtained data were evaluated by applying an analysis of variance (ANOVA) and the least significant differences (LSD) test (*P* < 0.05). A principal component analyses (PCAs) were performed using the data for the CLPP indices, the AWCD data for the six groups in which the 31 carbon substrates of Biolog EcoPlates™ were grouped and the data of enzyme activities. In addition, the analyses of the PC scores using the three-way and two-way MANOVA were also performed. All of the statistical analyses were performed using the Statistica 12.0 PL software package. Detailed information related to the analysis and interpretation of results are presented in Supplementary Materials.

## Results

### Community-level physiological profile (CLPP)

**Figure 1:**
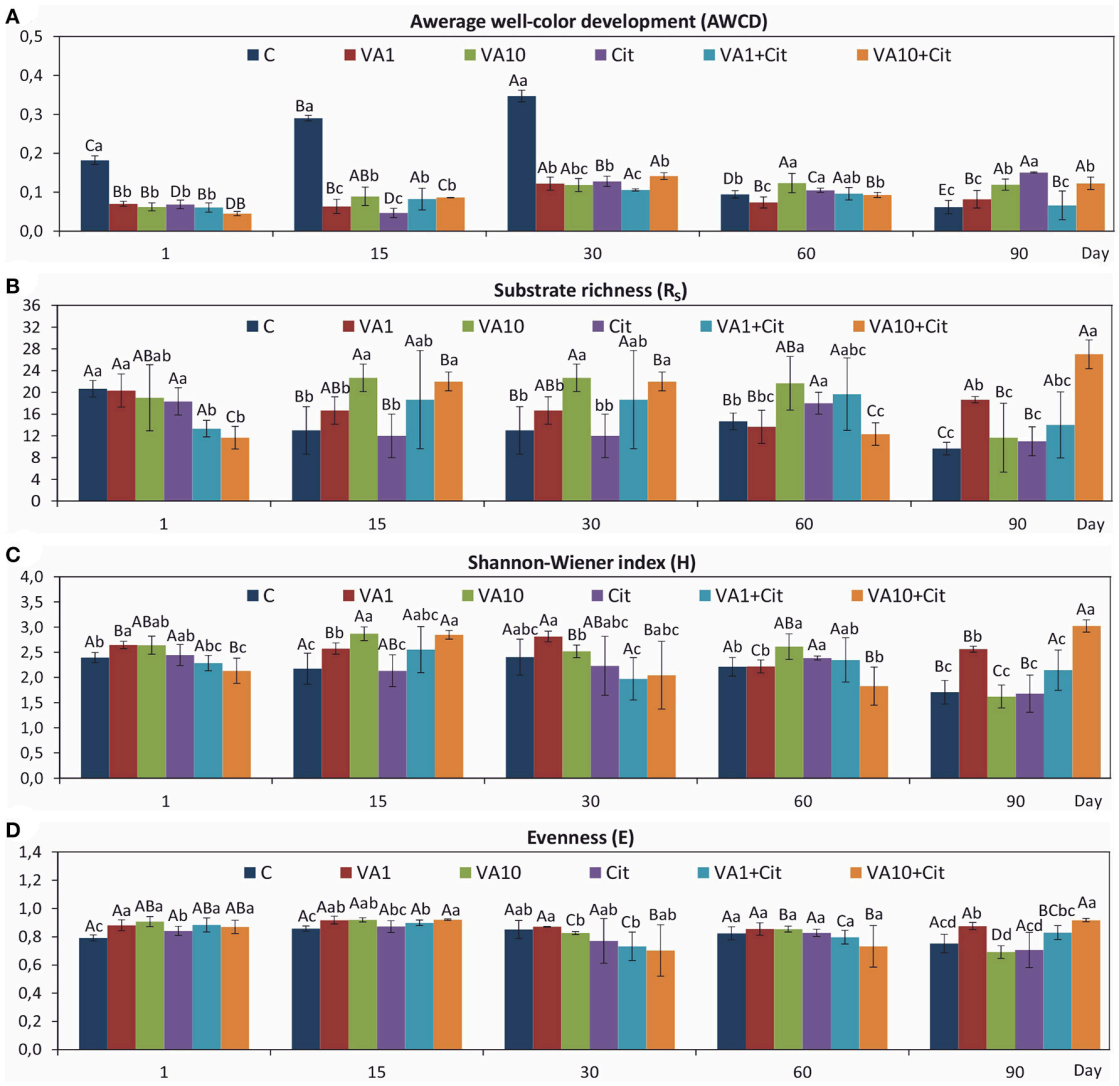
Values of the CLPP indices: AWCD **(A)**, R_S_ **(B)**, H **(C)**, and E **(D)** for the soil with VA and/or *C*. *freundii* obtained during the experimental period. C, control; VA1, 1 mg VA/kg soil; VA10, 10 mg VA/kg soil; Cit, *C*. *freundii*; VA1+Cit, 1 mg VA/kg soil + *C*. *freundii;* VA10+Cit, 10 mg VA/kg soil + *C*. *freundii*. The data are the means with standard deviations (*n* = 3). Different lowercase and uppercase letters within the values of each index indicate significant differences between treatments at the same sampling time and between sampling times within the same treatment (LSD *post hoc* test; *P* < 0.05), respectively.

The obtained CLPPs showed that there were significant differences in the values of the AWCD (Figure 1A), R_S_ (Figure 1B), H (Figure 1C), and E (Figure 1D) indices between the soil treated with VA and/or *C*. *freundii* and the control soil during the 90-day incubation. A significant decrease (*P* < 0.05) in the AWCD values in a response to VA and/or *C. freundii* introduction was observed on days 1, 15, and 30 of the experiment. In turn, at the end of the incubation (day 90), a significant increase in the AWCD values was observed for the vancomycin (VA1 and VA1+Cit)- and *C*. *freundii*-treated soils, and these values were almost 2- and 2.5-fold higher as compared to the value for the non-treated control, respectively (Figure 1A). The ANOVA revealed that the dosage of VA, *C*. *freundii*, the time of incubation and the interaction between the factors tested had a significant impact (*P* < 0.001) on the AWCD value (Table S1). The study revealed that the R_S_ value was only affected by the VA treatment (Table S1) and, that in general, its increase was found as compared to the non-treated control during the experiment. However, a higher dose of VA (VA10+Cit) negatively affected the R_S_ value at the beginning of the experiment (day 1) (Figure 1B). In turn, the H index (Figure 1C) was affected by all of the factors tested (Table S1).

Evaluation of the resistance of the CLPP indices to VA and/or *C*. *freundii* showed that these factors affected the values of the RS index during the experimental period (Table 1). In general, the ANOVA revealed that the treatment, time, and interaction between the factors tested had a significant impact (*P* < 0.001) on the resistance of the CLPP indices (Table S2). The highest reduction in the values of the RS index was observed in the case of the AWCD (Table 1). However, on day 90, this decrease was related to the stimulatory effect of VA and/or *C*. *freundii* on AWCD (Figure 1A). In the case of the remaining CLPP indices, a decrease in the values of the RS index, which was observed for some soil treatments (Table 1), was generally associated with the stimulatory effect of antibiotic and/or *C*. *freundii* during the experimental period (Figure 1). Calculation of the RL index at the end of the experiment (day 90) revealed that its value was different for each CLPP index. The positive values of the RL index for all of the soil treatments were obtained in relation to the AWCD (Table 2).

**Table 1:** Values of the resistance (RS) index for measured parameters obtained for each day of the experiment.

**Parameter**	**Day**	**Treatment**					**$\bar{x}$**
		**VA1**	**VA10**	**Cit**	**VA1+Cit**	**VA10+Cit**	
AWCD (average)	1	0.239^Ba^	0.206^Ba^	0.231^Ba^	0.199^Ca^	0.140^Ba^	0.203^B^
	15	0.123^Ba^	0.182^Ba^	0.087^Ba^	0.166^Ca^	0.175^Ba^	0.147^B^
	30	0.213^Ba^	0.205^Ba^	0.226^Ba^	0.180^Ca^	0.256^Ba^	0.216^B^
	60	0.634^Ac^	0.553^Ac^	0.800^Ab^	0.814^Ab^	0.951^Aa^	0.750^A^
	90	0.504^Aa^	0.020^Cb^	−0.187^Cc^	0.623^Ba^	−0.008^Bb^	0.191^B^
Substrate richness (R_S_)	1	0.896^Aa^	0.710^Aab^	0.796^Aa^	0.475^Ab^	0.392^Bb^	0.654^A^
	15	0.530^Bab^	0.128^Bc^	0.858^Aa^	0.479^Abc^	0.166^Bc^	0.432^AB^
	30	0.530^Bab^	0.128^Bc^	0.858^Aa^	0.479^Abc^	0.166^Bc^	0.432^AB^
	60	0.829^ABa^	0.381^ABb^	0.630^Aa^	0.567^Aa^	0.722^Aa^	0.626^A^
	90	0.034^Cb^	0.437^ABa^	0.769^Aa^	0.443^Aa^	−0.285^Cb^	0.280^B^
Shannon-Wiener index (H)	1	0.810^ABa^	0.818^ABa^	0.930^Aa^	0.911^Aa^	0.803^Aa^	0.854^A^
	15	0.686^Bbc^	0.512^Cc^	0.961^Aa^	0.711^ABCb^	0.524^Ac^	0.679^BC^
	30	0.704^Ba^	0.847^ABa^	0.832^Aa^	0.691^BCa^	0.738^Aa^	0.762^AB^
	60	0.964^Aa^	0.696^BCb^	0.853^Aab^	0.831^ABab^	0.705^Ab^	0.810^AB^
	90	0.331^Cc^	0.904^Aa^	0.888^Aa^	0.603^Cb^	0.127^Bd^	0.571^C^
Evenness (E)	1	0.797^Aa^	0.744^Ba^	0.881^ABa^	0.792^Ba^	0.823^ABa^	0.808^B^
	15	0.870^Aa^	0.866^ABa^	0.959^Aa^	0.908^ABa^	0.863^Aa^	0.893^A^
	30	0.894^Aab^	0.906^Aa^	0.810^Babc^	0.753^Bbc^	0.712^BCc^	0.815^B^
	60	0.927^Aab^	0.930^Aab^	0.964^Aa^	0.935^Aab^	0.794^Bb^	0.910^A^
	90	0.716^Bb^	0.853^ABab^	0.861^ABa^	0.812^ABab^	0.637^Cb^	0.776^B^
AWCD–amines	1	0.757^Aa^	0.281^Ab^	0.414^Ab^	0.282^Ab^	0.224^Ab^	0.392^A^
	15	0.065^BCb^	0.422^Aa^	0.143^Aab^	0.230^Aab^	0.272^Aab^	0.226^AB^
	30	0.249^Bb^	0.251^ABb^	0.212^Ab^	0.179^Ab^	0.647^Aa^	0.308^A^
	60	−0.004^BCbc^	−0.015^Bcbc^	−0.325^Bc^	0.185^Aab^	0.368^Aa^	0.042^BC^
	90	−0.148^Ca^	−0.067^Ca^	0.105^Aa^	0.077^Aa^	−0.535^Bb^	−0.114^C^
AWCD–amino acids	1	0.382^ABb^	0.870^Aa^	0.291^ABbc^	0.245^ABbc^	0.024^Bc^	0.362^A^
	15	0.181^ABa^	0.137^Ca^	0.054^Ba^	0.080^Ba^	0.147^Ba^	0.120^B^
	30	0.101^Ba^	0.036^Ca^	0.250^ABa^	0.059^Ba^	0.042^Ba^	0.097^B^
	60	0.489^Aa^	0.071^Cb^	0.582^Aa^	0.537^Aa^	0.543^Aa^	0.444^A^
	90	−0.553^Cc^	0.480^Ba^	−0.885^Cc^	−0.121^Cb^	−0.757^Cc^	−0.367^C^
AWCD–carbohydrates	1	0.570^Aa^	0.810^Aa^	0.513^Aa^	0.792^Aa^	0.698^Aa^	0.676^A^
	15	0.202^ABa^	0.220^Ba^	0.102^Ba^	0.252^Ba^	0.232^Ba^	0.202^B^
	30	0.117^Ba^	0.095^Ba^	0.051^Ba^	0.050^Ba^	0.064^BCa^	0.075^BC^
	60	0.274^ABa^	−0.371^Cc^	−0.119^Cab^	−0.418^Cc^	−0.256^Cbc^	−0.178^C^
	90	−0.222^Ca^	0.040^Ba^	0.014^Ba^	−0.120^Ba^	−0.526^Cb^	−0.163^C^
AWCD–carboxylic acids	1	0.305^Aa^	0.300^Ba^	0.344^ABa^	0.143^Ba^	0.162^Aa^	0.251^B^
	15	0.180^Aa^	0.225^Ba^	0.053^Ba^	0.237^Ba^	0.175^Aa^	0.174^B^
	30	0.309^Aab^	0.179^Bb^	0.215^Bb^	0.602^Aa^	0.128^Bb^	0.286^B^
	60	0.396^Abc^	0.646^Aab^	0.572^Aab^	0.739^Aa^	0.165^Ac^	0.504^A^
	90	−0.636^Bb^	−0.566^Cb^	−0.734^Cb^	−0.230^Ca^	−0.799^Bb^	−0.593^C^
AWCD–miscellaneous	1	0.091^Ba^	0.095^Ba^	0.255^Ba^	0.193^Ba^	0.100^Da^	0.146^B^
	15	0.119^Ba^	0.145^Ba^	0.160^Ba^	0.222^Ba^	0.172^Ca^	0.164^B^
	30	0.583^Ab^	0.676^Aab^	0.814^Aa^	0.550^Ab^	0.319^BCc^	0.588^A^
	60	0.583^Ab^	0.574^Ab^	0.814^Aa^	0.639^Aab^	0.518^Bb^	0.626^A^
	90	0.576^Ab^	0.569^Ab^	0.894^Aa^	0.630^Aab^	0.824^Aa^	0.699^A^
AWCD–polymers	1	0.107^Ba^	0.137^Ba^	0.068^Ba^	0.081^Aa^	0.084^Ca^	0.096^B^
	15	0.052^Ba^	0.064^Ba^	0.002^Ba^	0.042^Aa^	0.100^Ca^	0.052^B^
	30	0.099^Bb^	0.170^Bb^	0.063^Bb^	0.059^Ab^	0.501^Aa^	0.178^AB^
	60	0.184^Bb^	0.717^Aa^	0.425^Ab^	0.205^Ab^	0.202^BCb^	0.346^A^
	90	0.458^Ab^	−0.516^Ca^	−0.650^Ca^	0.227^Ab^	0.423^ABb^	−0.011^B^
DHA activity	1	0.800^Ba^	0.650^Db^	0.817^Ba^	0.685^Bb^	0.517^Dc^	0.694^D^
	15	0.972^Aa^	0.794^Cb^	0.812^Bb^	0.970^Aa^	0.655^Cc^	0.841^C^
	30	0.967^Aa^	0.858^Bb^	0.786^Bc^	0.936^Aa^	0.740^Bc^	0.857^BC^
	60	0.969^Aa^	0.943^Aa^	0.955^Aa^	0.958^Aa^	0.783^Bb^	0.922^AB^
	90	0.954^Aa^	0.934^Aa^	0.940^Aa^	0.951^Aa^	0.975^Aa^	0.951^A^
PHOS-H activity	1	0.921^Aa^	0.780^Cc^	0.944^Ba^	0.844^Bb^	0.728^Dd^	0.843^C^
	15	0.992^Aa^	0.867^Bb^	0.971^Aa^	0.866^Bb^	0.888^Bb^	0.917^AB^
	30	0.975^Aa^	0.786^Cc^	0.931^BCb^	0.981^Aa^	0.679^Ed^	0.870^BC^
	60	0.993^Aa^	0.811^Cb^	0.991^Aa^	0.987^Aa^	0.794^Cb^	0.917^AB^
	90	0.964^Aa^	0.949^Aab^	0.904^Cc^	0.982^Aa^	0.942^Ab^	0.948^A^
PHOS-OH activity	1	0.983^Aa^	0.860^Bc^	0.911^Abc^	0.979^Ba^	0.887^Bc^	0.924^AB^
	15	0.965^Aa^	0.855^Bb^	0.978^Aa^	0.943^Ba^	0.824^BCb^	0.913^B^
	30	0.957^Aa^	0.845^Bb^	0.977^Aa^	0.957^Ba^	0.819^Cb^	0.911^B^
	60	0.960^Aa^	0.751^Cb^	0.966^Aa^	0.993^Aa^	0.710^Dc^	0.876^B^
	90	0.963^Aa^	0.978^Aa^	0.946^Aa^	0.966^ABa^	0.997^Aa^	0.971^A^
URE activity	1	0.806^Aab^	0.632^Cc^	0.822^Ca^	0.774^Bb^	0.582^Dd^	0.723^C^
	15	0.981^Aa^	0.568^Db^	0.972^ABa^	0.952^Aa^	0.521^Eb^	0.799^BC^
	30	0.956^Aa^	0.625^Cc^	0.936^BCa^	0.951^Aa^	0.671^Cb^	0.828^B^
	60	0.955^Aa^	0.688^Bc^	0.983^Aa^	0.946^Aa^	0.738^Bb^	0.862^AB^
	90	0.984^Aa^	0.948^Aab^	0.939^ABb^	0.937^Ab^	0.958^Aab^	0.953^A^

*The data are the means (n = 3). Different lowercase and uppercase letters within the values of each parameter indicate significant differences between treatments at the same sampling time and between sampling times within the same treatment (LSD post hoc test; P < 0.05), respectively. The values marked in gray or green indicate a significant inhibition or stimulation in relation to the non-treated soil, respectively. The explanation of the treatment abbreviations is given in Figure 1*.

**Table 2:** Values of the resilience (RL) index for measured parameters obtained at the end of the experiment.

**Parameter**	**Treatment**					**$\bar{x}$**
	**VA1**	**VA10**	**Cit**	**VA1+Cit**	**VA10+Cit**	
AWCD (average)	0.696^a^	0.351^b^	0.122^c^	0.788^a^	0.385^b^	0.468
Substrate richness (R_S_)	−0.785^b^	−0.066^ab^	0.276^a^	0.330^a^	−0.315^ab^	−0.112
Shannon-Wiener index (H)	−0.542^c^	0.460^a^	−0.003^b^	−0.556^c^	−0.681^c^	−0.264
Evenness (E)	−0.140^ab^	0.328^a^	0.072^ab^	0.077^ab^	−0.341^b^	−0.001
AWCD–amines	−0.429^b^	0.305^a^	0.313^a^	0.396^a^	−0.158^b^	0.085
AWCD–amino acids	−0.067^b^	0.165^ab^	−0.647^c^	0.479^a^	−0.065^b^	−0.027
AWCD–carbohydrates	−0.114^bc^	0.133^ab^	0.537^a^	−0.337^bc^	−0.622^c^	−0.081
AWCD–carboxylic acids	−0.096^ab^	0.043^ab^	−0.329^b^	0.481^a^	−0.300^b^	−0.040
AWCD–miscellaneous	0.715^a^	0.717^a^	0.887^a^	0.708^a^	0.889^a^	0.783
AWCD–polymers	0.877^a^	0.117^b^	−0.044^c^	0.774^a^	0.857^a^	0.516
DHA activity	0.723^a^	0.773^a^	0.621^a^	0.812^a^	0.940^a^	0.774
PHOS-H activity	0.497^a^	0.715^a^	−0.110^b^	0.851^a^	0.736^a^	0.538
PHOS-OH activity	−0.235^b^	0.788^a^	0.387^ab^	−0.041^b^	0.995^a^	0.380
URE activity	0.875^a^	0.815^a^	0.607^a^	0.623^a^	0.868^a^	0.757

*The data are the means (n = 3). Different letters within the values of each parameter indicate significant differences between treatments (LSD post hoc test; P < 0.05). The explanation of the treatment abbreviations is given in Figure 1*.

**Figure 2:**
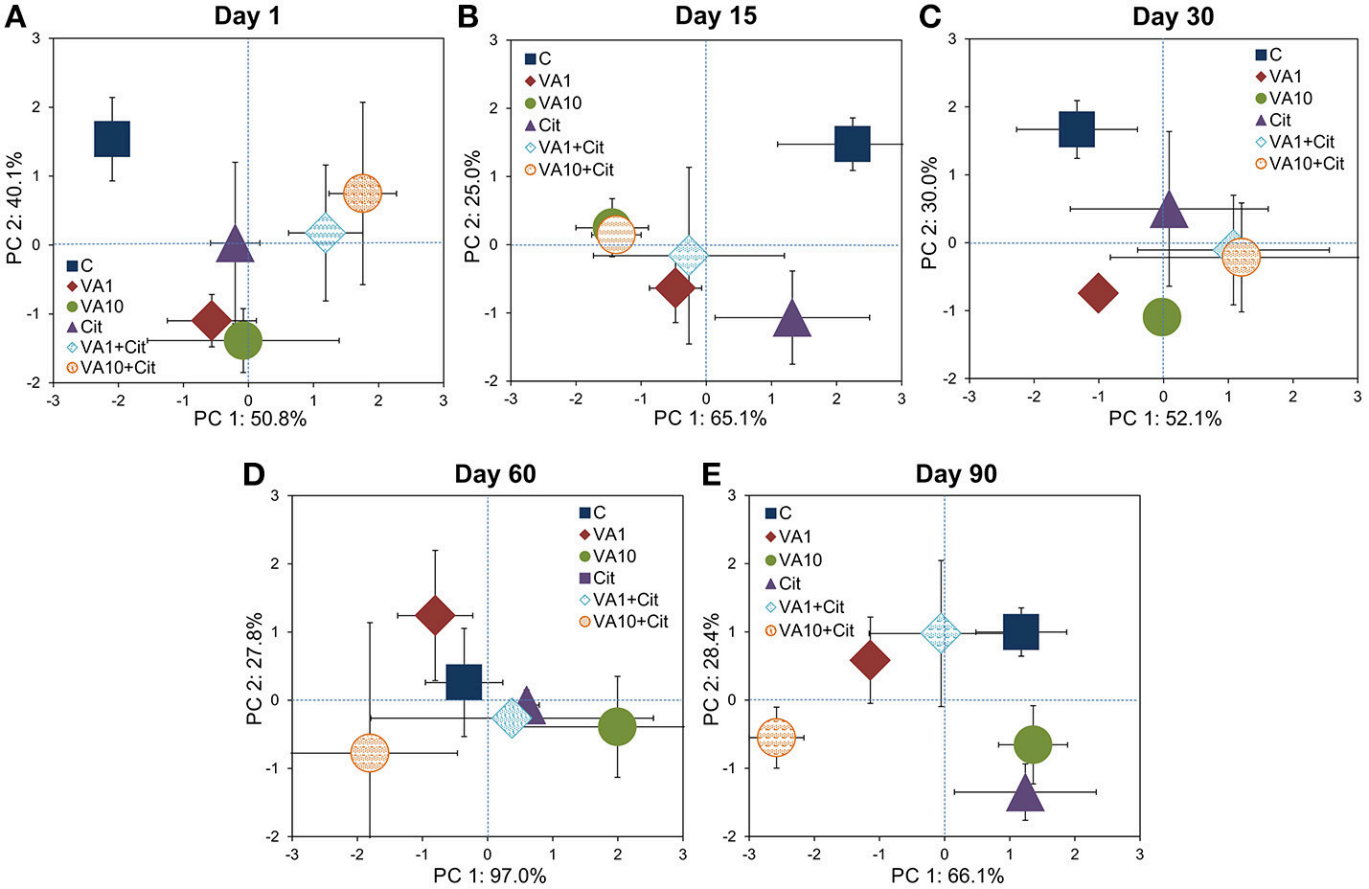
Results of the PCA analysis based on the CLPP indices on days 1 **(A)**, 15 **(B)**, 30 **(C)**, 60 **(D)**, and 90 **(E)**. The explanation of the treatment abbreviations is given in Figure 1.

The PCA plot obtained for the CLPPs (based on the AWCD, R_S_, H, and E values) for all of the sampling days revealed that samples were mainly scattered along the PC1 axis (Figure S1) and the pattern of variability depended on the bacterial strain, VA dosage and time (Table S3). The PCA plots obtained for each sampling day (Figure 2) showed a significant impact of the VA concentration and/or *C*. *freundii* on the CLPPs and it was evident on days 1, 15, 30, and 90 of the experiment (Table S4).

### Pattern of the utilization of carbon substrate groups

**Figure 3:**
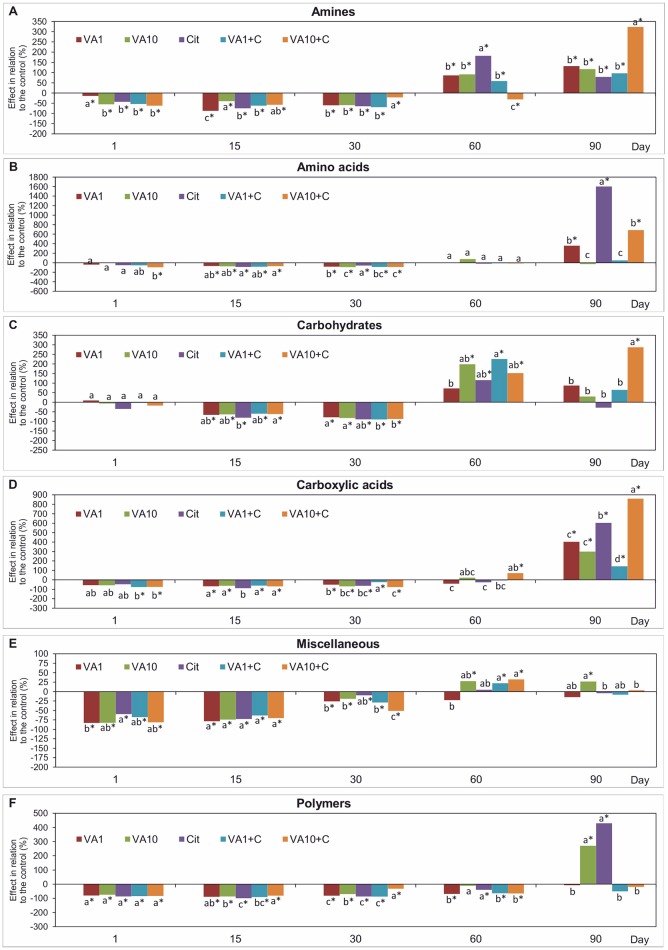
Effect of VA and/or *C*. *freundii* on the AWCD of the carbon substrate groups: amines **(A)**, amino acids **(B)**, carbohydrates **(C)**, carboxylic acids **(D)**, miscellaneous **(E)**, and polymers **(F)** during the experimental period. The data are the means (*n* = 3) and expressed as a percentage of the inhibition or stimulation in relation to the control soil. Different letters and asterisks within the values of each substrate indicate significant differences between treatments, and between treatments and control at the same sampling time (LSD *post hoc* test; *P* < 0.05), respectively. The explanation of the treatment abbreviations is given in Figure 1.

Analysis of the AWCD values for the six groups of 31 carbon substrates of the Biolog EcoPlates™ showed differences between the samples with VA and/or *C*. *freundii* and the non-treated soil (Figure 3). The obtained results generally indicated that all of the soil treatments contributed to a significant decrease (*P* < 0.05) in the AWCD values for the utilization of amines, miscellaneous and polymers in the first 30 days of the incubation. The AWCD values for the utilization of amino acids, carbohydrates and carboxylic acids were similar to those that were calculated for the non-treated soil on day 1. However, a negative effect of all of the soil treatments on the utilization of these carbon substrates groups was detected on days 15 and 30. At the next sampling times (days 60 and 90), all of the substrate usage patterns had recovered and, in many cases were higher (up to 1.3-17-fold) than those found in the control (Figure 3). A multivariate analysis showed that the utilization pattern of each substrate group was affected by the concentration of VA, the *C*. *freundii* strain and the time of incubation (Table S5).

Mean values of the RS index calculated for all of the soil treatments demonstrated that among the substrates, the highest resistance was observed for miscellaneous (0.445) while the lowest for carboxylic acids (0.124) (Table 1). In addition, the two-way ANOVA analysis generally revealed that VA and/or *C*. *freundii* significantly affected the values of the RS index for the utilization of carbon substrate groups during the experimental period (Table S2). Determination of the RL index at the end of the experiment (day 90) revealed that its value was different for the AWCD for each substrate group (Table 2). The positive values of the RL index for most of the soil treatments were obtained in relation to the AWCD for amines, miscellaneous, and polymers. In contrast, the RL index was found to be negative in the case of the AWCD for amino acids, carbohydrates and carboxylic acids (Table 2).

**Figure 4:**
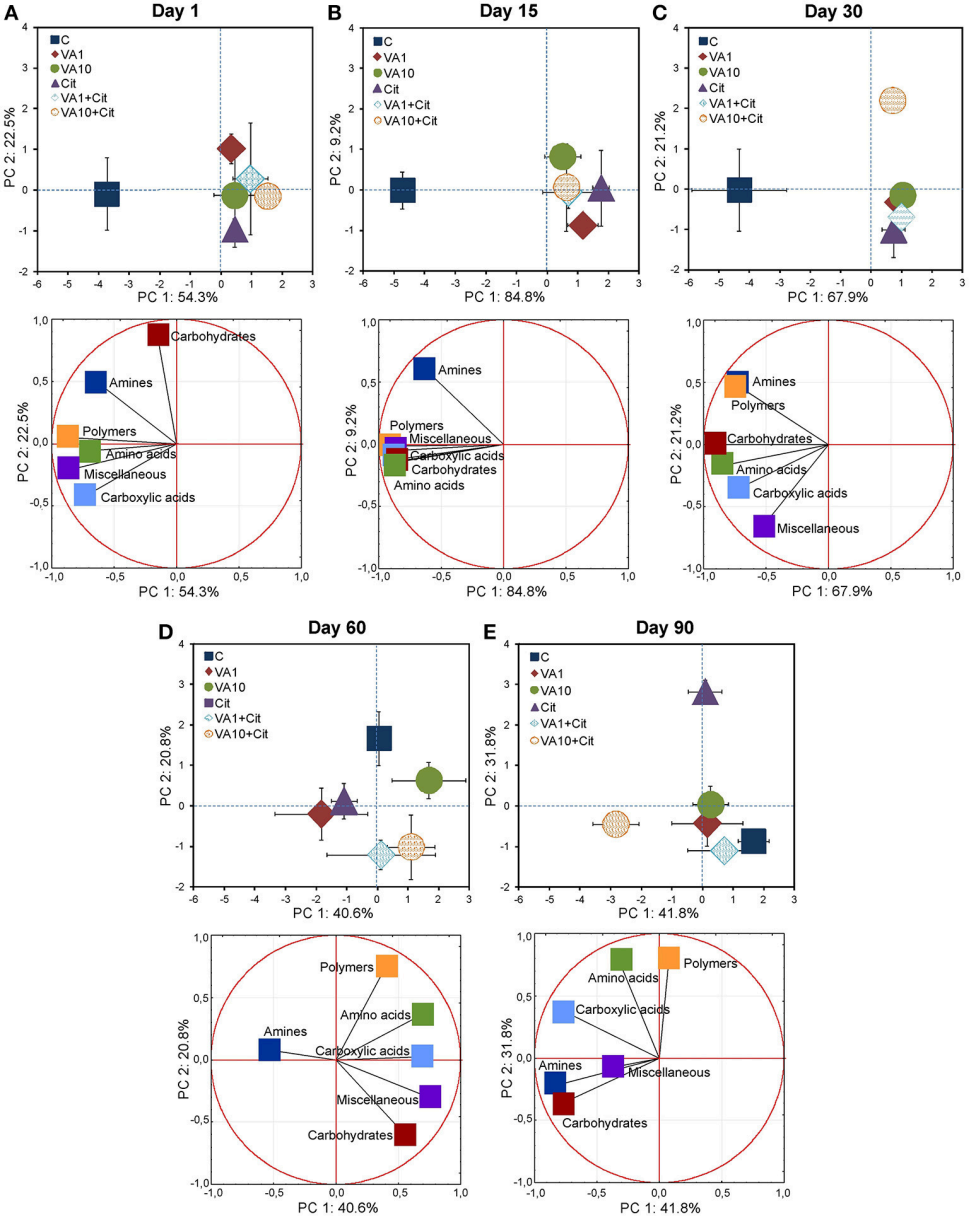
Results of the PCA analysis based on the data of the carbon substrate groups on days 1 **(A)**, 15 **(B)**, 30 **(C)**, 60 **(D)**, and 90 **(E)**. The explanation of the treatment abbreviations is given in Figure 1.

The PCA plots obtained from the AWCD values for the six groups of substrates for all of the sampling days (Figure S2) and for individual sampling days (Figure 4) revealed a pattern of variability depended on the bacterial strain, VA dosage and time (Table S6), and the bacterial strain and VA dosage (Table S7), respectively. Generally, a significant impact was evident on days 1, 15, and 30 of the experiment and, the carbon substrate utilization patterns for the treated soil samples separated from those obtained for the control soil (Figure 4 and Figure S2).

### Activity of enzymes

**Figure 5:**
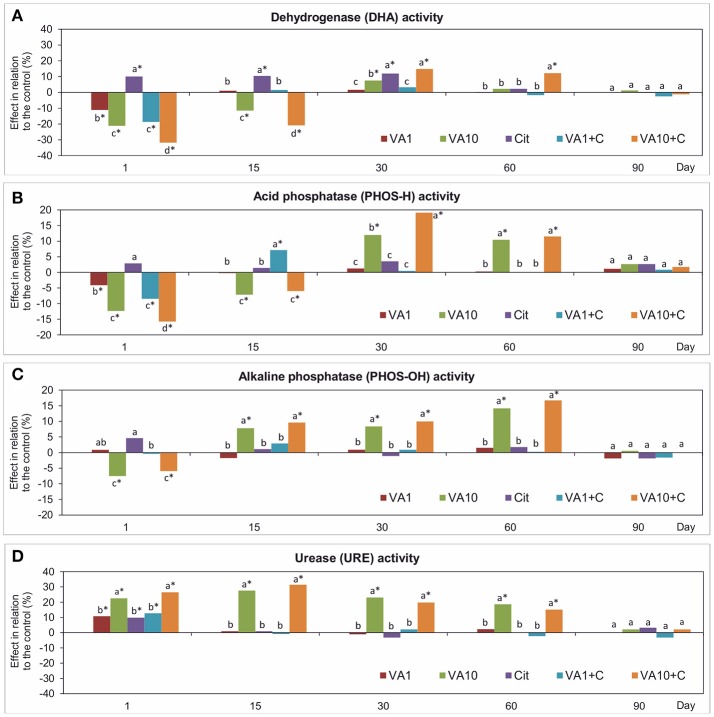
Effect of VA and/or *C*. *freundii* on the DHA **(A)**, PHOS-H **(B)**, PHOS-OH **(C)**, and URE **(D)** activity during the experimental period. The data are the means (*n* = 3) and expressed as a percentage of the inhibition or stimulation in relation to the control soil. Different letters and asterisks within the values of each enzyme indicate significant differences between treatments, and between treatments and control at the same sampling time (LSD *post hoc* test; *P* < 0.05), respectively. The explanation of the treatment abbreviations is given in Figure 1.

Analysis of the activity of enzymes showed differences between the samples with VA and/or *C*. *freundii* and the non-treated soil (Figure 5). The obtained results generally indicated that the higher soil treatments contributed to a significant decrease (*P* < 0.05) in the activity of DHA and PHOS-H, and PHOS-OH in the first 15 days and on day 1, respectively. In turn, a positive effect was detected on days 30 and 60. In the case of URE, an addition of the higher dose of VA and *C*. *freundii* stimulated its activity from day 1 up to day 60. On day 90, no effect was noticed in the soil samples with both doses of VA and/or *C*. *freundii* bacterial strain and the activity of all of the enzymes was similar to those determined for the non-treated soil (Figure 5). The three-way ANOVA revealed that different factors significantly influenced the activity of enzymes tested (Table S8).

The mean values of the RS index calculated for all of the soil treatments demonstrated that among the enzymes tested, the highest resistance was observed for PHOS-OH (0.919) while the lowest for URE (0.833) (Table 1). In addition, the two-way ANOVA analysis generally revealed that VA and/or *C*. *freundii* significantly affected the values of the RS index for the activity of enzymes during the experimental period (Table S2). Determination of the RL index at the end of the experiment (day 90) revealed that its mean value was found to be positive and reached the values of 0.774, 0.538, 0.380, and 0757 for DHA, PHOS-H, PHOS-OH, and URE, respectively (Table 2).

**Figure 6:**
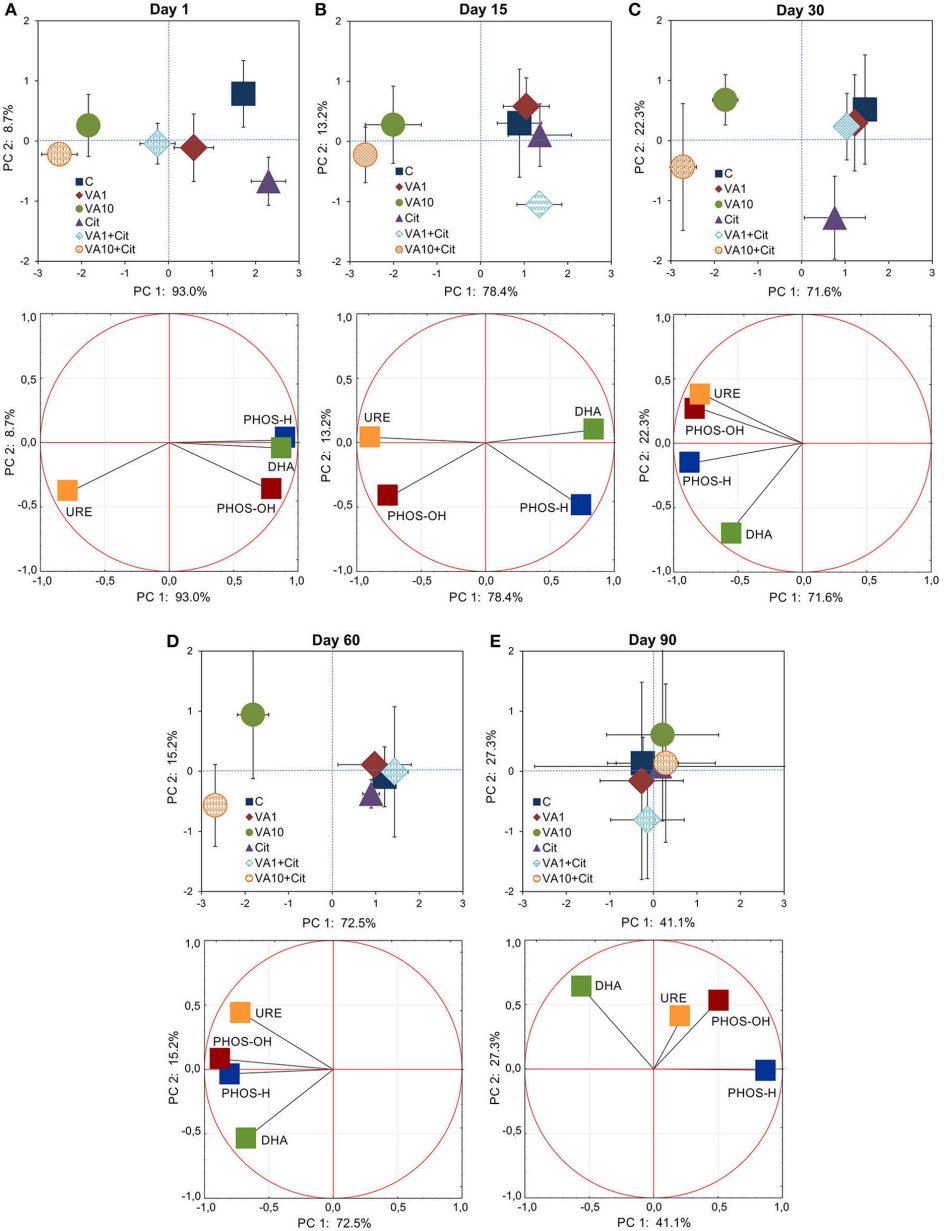
Results of the PCA analysis based on the data of the enzyme activities on days 1 **(A)**, 15 **(B)**, 30 **(C)**, 60 **(D)**, and 90 **(E)**. The explanation of the treatment abbreviations is given in Figure 1.

The PCA plots obtained from the enzyme activities for all of the sampling days (Figure S3) and for individual sampling days (Figure 6) revealed a pattern of variability depended on the bacterial strain, VA dosage and time (Table S9), and the bacterial strain and VA dosage (Table S10), respectively. Generally, a significant impact was evident up to day 60 of the experiment and, the enzyme activity patterns for the treated soil samples separated from those obtained for the control soil (Figure 6 and Figure S3).

### Dissipation of vancomycin in soil

**Figure 7:**
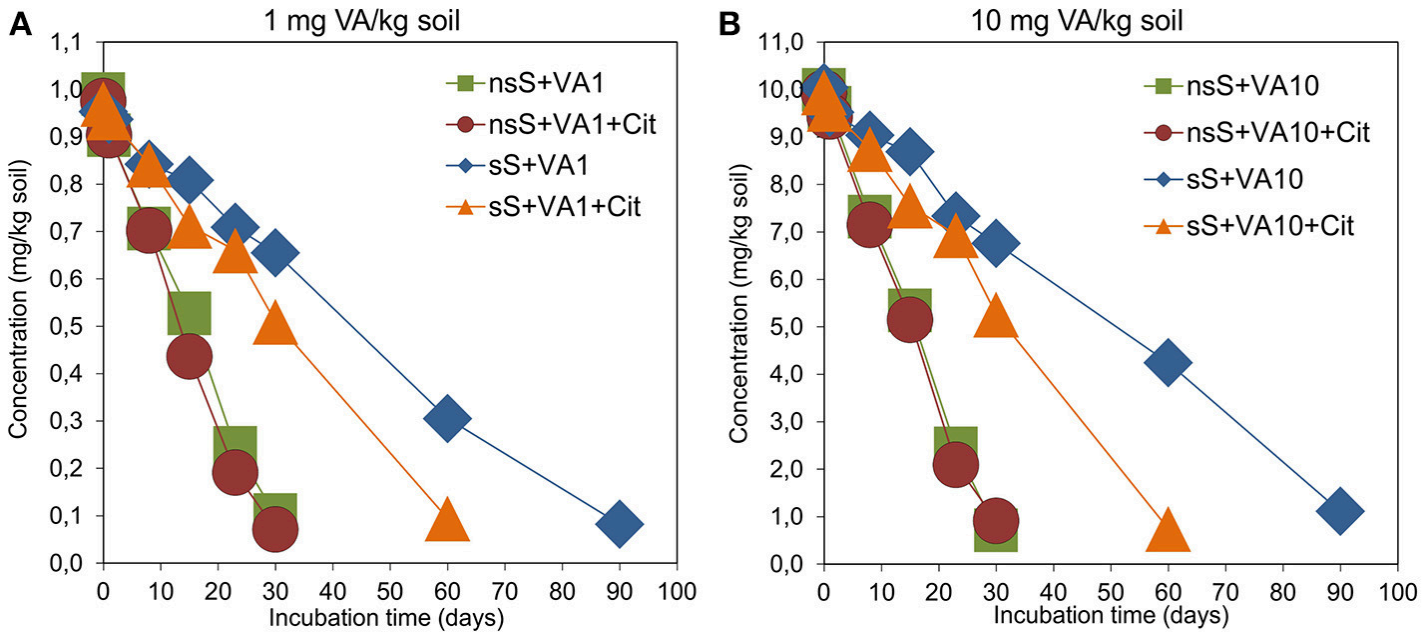
Dynamics of the VA dissipation in non-sterile (nsS) and sterile (sS) soils treated with VA and/or *C*. *freundii*. The explanation of the treatment abbreviations is given in Figure 1.

Based on the validation studies, the calibration curve was linear within a range of 0.005–20.0 μg/mL with *R*^2^ = 0.9997 (Figure S4). The limits of quantification (LOQ) and detection (LOD) as well as recoveries for VA were 0.01 mg/kg soil, 0.1 mg/kg soil and 87.2-102%, respectively (Table S11). Chromatograms for the VA standard, control and vancomycin-treated soil samples obtained during the validation studies are presented in Figures S5, S6. The results of the dissipation experiment of VA are presented in Figure 7. Our study showed that the dissipation of the antibiotic in VA1-, VA10-, VA1+Cit-, and VA10+Cit-treated nsS was relatively fast. Almost 100% of VA applied at both concentrations was degraded within 30 days of the experiment. There were no differences in the values of the DT50 of vancomycin between the treatments. The kinetic data indicated that the dissipation process followed zero-order kinetics and the calculated DT50 values were about 16 days for all of the non-sterile treatments (Table 3). The dissipation of VA in sS+VA and sS+VA10 was three times slower compared to the dissipation of the antibiotic in nsS. Both dosages of VA were almost completely degraded within 90 days and the DT50 values were 47.33 and 49.84 days, respectively (Table 3). Our study also showed that *C*. *freundii* had the potential to degrade VA and was able to degrade VA. In sS that had been inoculated with the *C*. *freundii* strain, the antibiotic was almost completely degraded within 60 days and the DT50 reached the values of 32.82 and 32.74 days for sS+VA+Cit and sS+VA10+Cit, respectively.

**Table 3:** The kinetic data of the disappearance of vancomycin in the soil.

**Treatment**	**Regression equation**	***R*^2^**	***k* (day)**	**DT_50_ (days)**
nsS+VA1	*C*_t_ −*C*_0_ = −0.0294*t* − 0.0349	0.9934	0.0296 ± 0.0023	15.64 ± 1.16^b^
nsS+VA10	*C*_t_ −*C*_0_ = −0.3132*t* − 0.0690	0.9980	0.3093 ± 0.0084	15.73 ± 0.72^b^
nsS+VA1+Cit	*C*_t_ −*C*_0_ = −0.0308*t* − 0.0338	0.9889	0.0301 ± 0.0043	14.73 ± 1.21^b^
nsS+VA10+Cit	*C*_t_ −*C*_0_ = −0.3085*t* − 0.1935	0.9923	0.3005 ± 0.0068	15.46 ± 0.92^b^
sS+VA1	*C*_t_ −*C*_0_ = −0.0098*t* − 0.0123	0.9946	0.0097 ± 0.0012	47.33 ± 1.46^a^
sS+VA10	*C*_t_ −*C*_0_ = −0.0963*t* − 0.2188	0.9949	0.0992 ± 0.0223	49.84 ± 1.76^a^
sS+VA1+Cit	*C*_t_ −*C*_0_ = −0.0144*t* − 0.0115	0.9956	0.0146 ± 0.0063	32.82 ± 0.58^c^
sS+VA10+Cit	*C*_t_ −*C*_0_ = −0.1516*t* − 0.0072	0.9949	0.1533 ± 0.0440	32.74 ± 0.89^c^

*sS, sterile soil; nsS, non-sterile soil; VA1, vancomycin (1 mg/kg soil); VA10, vancomycin (10 mg/kg soil); Cit, C. freundii. The data are the means with standard deviations (n = 3). Different letters within the DT50 values indicate significant differences between treatments (LSD post hoc test; *P* < 0.05)*.

The obtained results indicated that the dissipation of the antibiotic was independent of the concentration that had been used. This was confirmed by an ANOVA analysis, which revealed that the DT50 value was only affected by the type of soil (*P* < 0.001) and the *C*. *freundii* strain (*P* < 0.001; Table S12). The type of soil explained up to 83.1% of the variability. The concentration of VA had no effect (*P* = 0.801) on the DT50 value. A multivariate analysis also revealed that the DT50 value was only affected by the interaction between the type of soil and the *C*. *freundii* strain (*P* < 0.001) and that it explained 8.1% of the variability (Table S12).

## Discussion

Studies on the metabolic activity of microorganisms are of great significance as they show the biochemical potential of soils. Any toxicant application into soils that might affect the soil microorganism and their metabolic potential may generate changes in the productivity of soils (Badiane et al., 2001; Gil-Sotres et al., 2005; Liu et al., 2015; Cycoń et al., 2016b). In our previous study, using the DGGE and PLFA profiling, we found that application of VA and VA+*C. freundii* significantly altered the genetic and structural diversity of soil microbial communities (Cycoń et al., 2016a**)**. Surprisingly, we observed that the biomass of PLFAs characteristic for Gram-positive bacteria in the VA-treated soils was higher in comparison with the control. These data suggest that a large fraction of Gram-positive inhabiting tested soils was resistant to VA and reached a high biomass probably due to use of VA as the source of carbon. A high number of active metabolically Gram-positive bacteria allowed them to successfully competed with Gram-negative bacteria, for which a substantial decrease in the PLFA biomass was observed. However, the effect of VA on qualitative and quantitative changes within indigenous microbial communities was transient.

Results of this study demonstrated that alterations in the structure of soil bacterial assemblages were reflected in changes of the total microbial activity. We found that the AWCD values for samples treated with VA and/or *C*. *freundii* were significantly lower as compared to the control, however this effect was observed only during 30 days of the experiment. A short-lasting effect of various antibiotics on soil microbial activity was also observed by other authors. For example, Fang et al. (2014, 2016) reported that chlortetracycline applied at 1 and 10 mg/kg soil decreased the values of the AWCD for 35 days. From this day, irrespective of the frequency of antibiotic application, the AWCD values gradually recovered to the control level. A short-term detrimental effect of tetracycline on the community level physiological profiles was also observed by Chessa et al. (2016). The CLPP data showed that tested soil were susceptible to tetracycline applied at the concentration of 500 mg/kg for only 7 days. In contrast, Toth et al. (2011) showed that the application of chlortetracycline and sulfadimethoxine did not significantly change the CLPP parameters, whereas monensin slightly increased the value of the H index. In our study, alterations in the preferential degradation of some of the substrates in Eco-plates by microorganisms from VA– and VA+*C*. *freundii*-treated soil samples were found. VA impacts microorganisms by changing their ability to utilize different carbon sources, thus affecting the metabolic diversity of soil microbial communities. Similarly, Xu et al. (2016) found that a high level of sulfadiazine decreased the utilization rates of carbohydrate, carboxylic acid, amino acid, and aromatic acid. Also, sulfamethoxazole applied at concentrations of 100 and 1,000 mg/kg decreased the degradation of all substrates with the exception of the polymers (Pino-Otín et al., 2017). A significant decrease in the utilization of some substrates (carbohydrates and miscellaneous) was also observed in soils treated with sulfamethoxazole (Liu F. et al., 2012). However, this short-term effect was only observed 7 days after the antibiotic application and on day 21, the utilization of the substrates increased compared to the first sampling day. In addition, Kong et al. (2006) observed that the substrate utilization pattern changed significantly with increasing concentrations of oxytetracycline. In contrast, doxycycline application had a general positive effect on the utilization of substrates (Wang et al., 2016).

Changes in the activity of soil microbial communities pointed by Biolog method were also proved by measuring the activities of selected enzymes. The application of VA (at a higher concentration) and/or *C*. *freundii* significantly shifted the pattern of the enzyme activities. A short-term negative effect of VA manifested toward the activity of DHA, PHOS-H, and PHOS-OH. Similar to our results, a temporary decrease in DHA in soil treated with oxytetracycline or lincomycin (both at 50 and 200 mg/kg soil) was observed by Unger et al. (2013). The same effect was found in soil treated with manure containing sulfamethazine (Pinna et al., 2012). An inhibition of DHA activity along the gradient of the oxytetracycline concentration over the entire experimental period was found by and Chen et al. (2013). In another study, the activity of DHA in chlortetracycline-treated soils (1, 10 and 100 mg/kg) increased on the first day but then significantly decreased for up to 45 days (Liu et al., 2015). In our study, VA and VA+*C. freundii* increased the activity of all the enzymes on days 30 and 60 in comparison with the control. However, lower activities of both phosphatases were observed in VA-treatments at the beginning of the experiment. Similarly, six different antibiotics, i.e., chlortetracycline, tetracycline, tylosin, sulfamethoxazole, sulfamethazine and trimethoprim when applied at dosages of 1–300 mg/kg soil inhibited the PHOS-H activity during the experiment, although this effect was slight (Liu et al., 2009, 2015). The study by Yang et al. (2009) revealed that among the PHOSs tested, only PHOS-OH was sensitive to the application of oxytetracycline with a 41.3% decline in the activity of enzyme at a dosage of 10 mg/kg soil and, a further decrease of 64.3–80.8% when concentration of the antibiotic exceeded 30 mg/kg. In contrast, Ma et al. (2016**)** found that oxytetracycline had no effect on the soil neutral PHOS activity over a 120-day incubation period even when large amounts of the antibiotic were applied (up to 30 mg/kg). An analysis of URE activity in soil that had been treated with vancomycin and/or *C*. *freundii* showed stimulated stimulation of this enzyme for up to 60 days. In contrast, in oxytetracycline- and tetracycline-treated soils, the URE activity was significantly inhibited along the gradient of concentrations of the antibiotics (Wei et al., 2009; Chen et al., 2013). In other studies, the URE activity in the soil was slightly affected by sulfadiazine (Hammesfahr et al., 2011) or was affected for a short time by sulfamethazine (Pinna et al., 2012). Our results indicated that among the enzymes tested, DHA was the most sensitive to the application of the antibiotic. The low activity of DHA in the soils that had been treated with VA at the beginning of the experiment may be related with the death or inhibition of some microorganism that is sensitive to antibiotics and that is responsible for the production of enzymes. In addition, the DHAs that are released from dead microorganisms do not accumulate in soils since they are rapidly degraded (Alef, 1995). In turn, PHOSs and URE are more stable in soils because they are immobilized by various soil compounds (Gianfreda et al., 1994).

In addition to a higher activity of enzymes in higher VA dosage-treated soils (especially between 30 and 60 days), an increase of the AWCD values was observed, suggesting enhancement of the total catabolic potential of soil. This effect might be result from the ability of microorganisms to use an antibiotic as the additional compounds for their growth. This explanation might be supported by the fast disappearance of VA in soil used in our study. Moreover, the most of the inoculants do not survive for a long time in the bioaugmented soil, and nutrients released from dead cell provide an additional source of carbon and energy. The pool of nutrients might be also extended by compounds originated from sensitive bacteria killed by antibiotic applied into the soil (Pinna et al., 2012; Ding et al., 2014; Chessa et al., 2016). A consequence of these above phenomena may be the increase in the microbial biomass and enzyme production (Westergaard et al., 2001; Hammesfahr et al., 2011). In general, a multivariate analysis revealed that the inoculation of *C. freundii* had no significant effect on the metabolic potential of soil microbial communities with the exception of the short-term stimulation of DHA, PHOSs and URE. This showed that inoculants, in contrast to VA, did not exert a stressful condition for indigenous microorganisms. Moreover, introduced bacteria did not modify the action of VA. The lack of changes might also be related to the competition with autochthonic microorganisms. In addition, the inhibition *via* compounds that are synthesized by these organisms may also be taken into consideration. Moreover, the introduction of inoculants into soil is stressful resulting in the necessity of their adaptation to the new soil conditions (Karpouzas et al., 2000; Singh et al., 2006).

The short-term changes in the soil metabolic diversity in the response to VA and/or *C*. *freundii* application may be also related to the resilience and resistance of tested microbial communities. The RS and RL indices enable to check if microbial communities exposed to various stressors can remain stable and/or achieve the origin level of metabolic activity (Orwin and Wardle, 2004). Significant changes in the RS value were observed up to 60 days of the experiment for soil treated with a higher dose of VA (irrespectively of *C. fruendii* inoculation). URE and DHA were more sensitive to VA in comparison with phosphatases. A similar phenomenon was also previously demonstrated for soil that had been contaminated with pesticides (Baćmaga et al., 2015). However, in our study, there were no differences in the values of the RS and RL indices between the soil treatments on day 90 of the experiment. These results showed that intrinsic properties of soil microbial communities are a key mechanism driving functional stability of soil ecosystem (Song et al., 2015). Even if microbial populations are sensitive to perturbation, the entire community may be resilient and have the ability to return to its original activity (Allison and Martiny, 2008). Second, microorganisms in a new community may act differentially but the final metabolic outcome is similar to those observed in the non-disturbed community. Moreover, the resilience potential reflects the multifunctionality of the soil microorganisms (Ludwig et al., 2017). It has been thought that two mechanisms are responsible for the lack of changes in the ecosystem processes rate despite the alteration in the biochemical microbial diversity. First, a new community may contain microorganisms that are functionally redundant with microorganism that were affected by stressors. Second, microorganism in a new community may act differentially but the final level of biological processes is similar to those observed in non-disturbed community (Allison and Martiny, 2008).

The obtained results revealed that the dissipation of VA in nsS was relatively fast and independent of the antibiotic concentration. Based on the kinetic model, the DT50 values for the dissipation of VA at 1 and 10 mg/kg soil were 15.64 and 15.73 days. As proposed by Crane et al. (2010), antibiotics that are characterized by DT50 values of 5-22 days belong to the group of high degradability chemicals in soils. In this context, VA with a DT50 of about 16 days may be classified as a compound with a low persistence in soil. The relatively short-term persistence of VA in soil might also be related to its solubility in water and its low affinity to adsorption by various components of the soil (Thiele-Bruhn, 2003). Antibiotics are subjected to various processes in the soil such as volatilization, transformation or degradation, sorption-desorption, uptake by plants and transport into groundwater and surface waters (Accinelli et al., 2007; Lin and Gan, 2011; Yang et al., 2012; Manzetti and Ghisi, 2014; Awad et al., 2016; Pan and Chu, 2016; Topp et al., 2016). However, many factors such as the chemical structure, properties and concentration of an antibiotic, the physico-chemical properties of the soil, the microbial population and the incubation conditions play a major role in the degradation of antibiotics in soil. Antibiotics differ in their susceptibility to degradation in soil as was evidenced by the large range of the DT50 or half-life values between <1 and 3,466 days (Crane et al., 2010; Walters et al., 2010; Hammesfahr et al., 2011; Braschi et al., 2013; Awad et al., 2016).

Our study showed that the dissipation of VA in nsS was three times faster compared to the dissipation of antibiotics in sS as was indicated by the DT50 values. These results suggest that in addition to abiotic processes, degradation by microorganisms was the main mechanisms of VA disappearance in soil. Moreover, Pan and Chu (2016) showed that erythromycin (0.1 mg/kg soil) applied into clay loamy soil was degraded faster in nsS compared to sS with a DT50 of 6.4 and 40.8 days, respectively. Accinelli et al. (2007) reported that sulfachloropyridazine (10 mg/kg soil) was degraded almost three times faster in soils with autochthonous microorganisms (half-life 20–26 days) compared to sterile soils (half-life 68–71 days). (Zhang W. et al., 2017; Zhang Y. et al., 2017) also showed that microbial activity contributes in the biotransformation of sulfadiazine in soil, for which the calculated DT50 reached values of 8.48, 8.97, and 10.22 days (non-sterile soil) and 30.09, 26.55, and 21.21 days (sterile soil) for 4, 10, and 20 mg/kg, respectively. This phenomenon has also been found for other antibiotics (Lin and Gan, 2011; Srinivasan and Sarmah, 2014; Pan and Chu, 2016).

Our study also showed that *C*. *freundii* was characterized by a degradation potential in relation to the VA as showed by the disappearance of VA in sterile soil inoculated with this strain. In contrast, we did not observe an acceleration of VA dissipation in non-sterile soil by *C*. *freundii* and the DT50 values for VA were similar to those obtained for non-sterile soil without the strain. A lack of changes in the degradation rate of VA after the inoculation of *C*. *freundii* into the soil might be related to the ability of the inoculated strain to survive and its competition with autochthonic soil microorganisms or inhibition *via* compounds produced by these organisms (Karpouzas et al., 2000; Cycoń et al., 2014), which were not observed in sS. In turn, the study by Topp et al. (2016) revealed that the bioremediation potential of *Microbacterium* sp. increased the mineralization of sulfamethazine by 44–57% in an agricultural soil. Other studies have confirmed that several bacteria isolated from antibiotic-contaminated sources (i.e., patients, soil, sediments, and sludge) belonging to different genera were capable of degrading antibiotics in liquid cultures (Xin et al., 2012; Topp et al., 2013; Leng et al., 2016; Mulla et al., 2018; Wen et al., 2018).

## Conclusions

The decrease in the activity of soil microorganisms found in this study was consistent with results of our previous experiments dealing with changes in the structural and genetic diversity of a microbial community in a response to the application of VA and/or *C. freundii*. As was shown by the degradation data, VA was almost completely degraded in nsS within 30 days. At that time, we also observed a decrease in the metabolic activity of soil microorganism as was indicated by the data from CLPP and enzyme activities. On the next sampling days, no effects or stimulation of microbial activity were found. These results suggest that as long as VA was present in the soil, it negatively affected microbial activity. Regardless if VA was applied alone or with *C. freundii* it altered the catabolic potential of soil thus created the stressful conditions for autochthonic microbes. In turn, *C. freundii* introduced into soil alone did not pose a threat for metabolic activity of microbial communities. An analysis of the RS and RL indices showed that there were differences in the resistance and resilience of measured activities to disturbances caused by antibiotic and/or *C*. *freundii*. The loss of the ability of microbial communities to degrade some substrates and the decreasing activity of soil enzymes in VA-treated soil may be connected with the inhibition of some microorganisms, which are responsible for the production of certain enzymes. However, the processes of the selection and adaptation of microorganisms to antibiotics as well their functional redundancy are responsible for the recovery of microbial communities from disturbances that are caused by vancomycin. Although the negative effect of VA on the metabolic pattern of soil microorganism was transient, the application of VA into soil may temporarily pose a potential risk for soil functioning. It seems that deeper understanding of mechanisms involved in the response of soil microorganisms to disturbances is crucial for the assessment of the impact of stressors on the soil function.

## Author contributions

KO and MC Conceived and designed experiments. KO, MC, AM, AZ, JS-D, and JB-W Contributed reagents and materials, performed experiments. KO, MC, AM, AZ, TW, and ZP-S Analyzed results. KO, MC, and ZP-S Wrote the paper.

### Conflict of interest statement

The authors declare that the research was conducted in the absence of any commercial or financial relationships that could be construed as a potential conflict of interest. The reviewer LAP and handling Editor declared their shared affiliation.
