# Supplementary material for: Vancomycin and/or Multidrug-Resistant Citrobacter Freundii Altered the Metabolic Pattern of Soil Microbial Community
Source: Front Microbiol. 2018 May 23;9:1047. doi: 10.3389/fmicb.2018.01047 (PMC5974218; doi:10.3389/fmicb.2018.01047)
Supplement: Supplementary file 1 [file Data_Sheet_1.PDF]

Supplementary Materials

Vancomycin and/or Multidrug-Resistant *Citrobacter freundii* Altered the Metabolic Pattern of Soil Microbial Community

Mariusz Cycoń[1,*], Kamila Orlewska[1], Anna Markowicz[2], Agnieszka Żmijowska[3], Joanna Smoleń-Dzirba[1], Jolanta Bratosiewicz-Wąsik[1], Tomasz J. Wąsik[1], Zofia Piotrowska-Seget[2]

[1] Department of Microbiology and Virology, School of Pharmacy with the Division of Laboratory Medicine, Medical University of Silesia, Sosnowiec, Poland,

[2] Department of Microbiology, University of Silesia, Katowice, Poland,

[3] Department of Ecotoxicology, Institute of Industrial Organic Chemistry, Pszczyna, Poland

***Correspondence:**

Mariusz Cycoń

mcycon@sum.edu.pl

MATERIALS AND METHODS

Design of Experiment

A loamy sand soil was used in the experiment. The properties of the soil were shown in a previous paper (**Cycoń et al., 2016a**) and determined according to suitable methods (**Cycoń et al., 2010**).

The experiment with non-sterile soil (nsS) and sterile (sS) soils had a completely randomized block design with the following treatments:

- nsS - control (C): 4,800 g (24 pots × 200 g soil); for analysis – 3 replicates × 5 sampling times
- nsS with vancomycin (1 mg/kg soil) – nsS+VA1: 4,800 g (24 pots × 200 g soil); for analysis – 3 replicates × 8 sampling times
- nsS with vancomycin (10 mg/kg soil) – nsS+VA10: 4,800 g (24 × 200 g); for analysis – 3 replicates × 8 sampling times
- nsS inoculated with *Citrobacter freundii* (1.6×10^7 cells/g soil) – nsS+Cit: 4,800 g (24 × 200 g); for analysis – 3 replicates × 5 sampling times
- nsS with vancomycin (1 mg/kg soil) and with *Citrobacter freundii* (1.6×10^7 cells/g soil) – nsS+VA1+Cit: 4,800 g (24 × 200 g); for analysis – 3 replicates × 8 sampling times
- nsS with vancomycin (10 mg/kg soil) and *Citrobacter freundii* (1.6×10^7 cells/g soil) – nsS+VA10+Cit: 4,800 g (24 × 200 g); for analysis – 3 replicates × 8 sampling times
- sS with vancomycin (1 mg/kg soil) – sS+VA1: 4,800 g (24 × 200 g); for analysis – 3 replicates × 8 sampling times
- sS with vancomycin (10 mg/kg soil) – sS+VA10: 4,800 g (24 × 200 g); for analysis – 3 replicates × 8 sampling times
- sS with vancomycin (1 mg/kg soil) and with *Citrobacter freundii* (1.6×10^7 cells/g soil) – sS+VA1+Cit: 4,800 g (24 × 200 g); for analysis – 3 replicates × 8 sampling times
- sS with vancomycin (10 mg/kg soil) and *Citrobacter freundii* (1.6×10^7 cells/g soil) – sS+VA10+Cit: 4,800 g (24 × 200 g); for analysis – 3 replicates × 8 sampling times

Soil samples (3 replicates for each treatment) were periodically removed (randomly) from the test system to evaluate the metabolic pattern and physiological diversity of a bacterial community (on days 1, 15, 30, 60 and 90) and the concentration of VA (on days 0, 1, 8, 15, 23, 30, 60 and 90).

In order to ensure an even distribution of the antibiotic in the soil, the VA solution was prepared in sterile pure water and then added to sterile quartz sand (< 0.5 mm). After evaporation of water in the dark, the mixture of sand (50 g/kg soil) and VA was added into the soil portion and thoroughly mixed. The concentrations of vancomycin that were used reflect the most adverse scenarios associated with the entry of large quantities of antibiotics into the soil as a result of the uncontrolled disposal of unused drugs into municipal waste or depositing them in landfills.

In order to prepare the inoculum, the bacterial strain was cultured in 200 mL Erlenmeyer flasks containing 100 mL of nutrient broth (BTL, Poland). At the exponential phase, the bacteria were pelleted by centrifugation (5 min, 10,000 *g*). The pellet was washed twice with 0.85% of sterile NaCl and then resuspended in 0.85% of sterile NaCl to obtain a bacterial suspension at a concentration of approximately 2.1×10^9 cells/mL. The cell density (OD 550 nm) was measured using a densitometer (Densimat®, bioMérieux, France). Next, the bacterial suspension was introduced into the soil in triplicate in order to produce a final bacterial count of approximately 1.6×10^7 cells/g soil.

The water content of the soils was adjusted to 50% of the maximum water holding capacity and checked every week. Throughout the incubation period, water losses exceeding 5% of the initial values were compensated for by the addition of sterile deionized water. The pots with soil samples were covered with perforated polypropylene sheets and were incubated in the dark at 22 ± 1°C for 90 days.

Analysis of the Community-Level Physiological Profile (CLPP)

Community level physiological profiles were assessed using the Biolog EcoPlate™ system (Biolog Inc., CA, USA). In order to extract the microbial community, soil suspensions (10 g dry weight of soil in 90 ml sterile 0.85% NaCl solution) were shaken for 1 h and then aliquots of 125 µl were inoculated onto plates, which were then incubated at 24°C in the dark. The readings were taken at 590 nm after inoculation and at 12 h intervals for 144 h using a BIOLOG™ microplate reader. The absorbance measurements for individual substrates (Table 1) were corrected against the control well, which contained only water, and by subtracting the absorbance of the first reading to eliminate the background absorbance (**Insam, 1997**).

Table 1. Six group of substrates involved in the CLPP analysis.

**Amines amides**	**Amino acids**	**Carbohydrates**
phenylethylamine	glycyl-L-glutamic acid	α-D-lactose
putrescine	L-arginine	ß-methyl-D-glucoside
	L-asparagine	D-cellobiose
	L-phenylalanine	D-mannitol
	L-serine	D-xylose
	L-threonine	i-erythritol
		N-acetyl-D-glucosamine
**Carboxylic acids**	**Miscellaneous**	**Polymers**
γ-hydroxybutyric acid	D,L-α-glycerol phosphate	α-cyclodextrin
α-ketobutyric acid	glucose-1-phosphate	glycogen
2-hydroxy benzoic acid	pyruvic acid methyl ester	Tween 40
4-hydroxy benzoic acid		Tween 80
D-galactonic acid α-lactone		
D-galacturonic acid		
D-glucosaminic acid		
D-malic acid		
itaconic acid		

Determination of Enzyme Activities

Dehydrogenase (DHA) activity was determined using 2,3,5-triphenyltetrazoliumchloride (TTC) as the substrate (0.5%, 5 mL) and incubating the soil samples (5 g) mixed with a Tris buffer (pH 7.6) (0.1M, 5 mL) at 25°C for 20 h. The produced triphenyl formazan (TPF) was extracted from the mixture with acetone and measured at 546 nm using a Jenway 6300 spectrophotometer (Jenway, UK). The activity of dehydrogenase was expressed as µg TPF/g/h (**Alef, 1995**).

Acid (PHOS-H) and alkaline (PHOS-OH) phosphatase activities were determined using *p*-nitrophenyl phosphate as the substrate (0.05 M, 1 mL). Soil samples (1 g) were mixed with a modified universal buffer (MUB) (15 mM, 4 mL) of pH 6.5 and 11 for acid and alkaline phosphatase assays, respectively, and the substrate solution and incubated for 1 h at 37°C. After incubation, $CaCl_2$ (0.5 M, 1 mL) and NaOH (0.5 M, 4 mL) were added to stop the reaction and to avoid the coloration caused by organic matter. The extracted *p*-nitrophenol (*p*-NP) was measured at 400 nm using a Jenway 6300 spectrophotometer (Jenway, UK). The activity of PHOS-H and PHOS-OH was expressed as µg *p*-NP/g/h (**Tabatabai and Bremner, 1969**).

Urease (URE) activity was determined using urea (10%, 10 mL) as a substrate, which was added to wet soil (10 g) supplemented with a citrate buffer (pH 6.7) (20 mL) followed by incubation at 37°C for 5 h. After incubation, the NH_4^+ concentrations in the reaction mixtures were determined. The analysis was based on the measurement of the intensity of the blue color yielded during the reaction of ammonium with sodium chlorate (0.9% active Cl_2) and sodium phenolate (phenol 50%, NaOH 21.6%, 1:1) at 630 nm using a Jenway 6300 spectrophotometer (Jenway, UK). The activity of URE was expressed as mg NH_4^+/kg/h (**Gianfreda et al. 1994**).

Analysis and Interpretation of Results

The CLPP in the soil samples were obtained using the Biolog® EcoPlate™ system (Biolog Inc., CA, USA) (**Insam, 1997**). Microbial activity, which is expressed as the average well-color development (AWCD), is a parameter that enables an integral picture of the differences in the utilization of carbon sources to be captured, was determined according to the equation (Eq. 1) (**Garland, 1997**).

$$AWCD = \sum OD_i/31 \quad (1)$$

where OD_i is the optical density value from each well.
Substrate richness (R_S) and the Shannon-Wiener index (H) (i.e. the richness and evenness of response) were calculated using an OD of 0.25 as the threshold for a positive response (**Garland, 1997**). The Shannon-Wiener index (H) and evenness (E) values were calculated according to the equations (Eq. 2) and (Eq. 3), respectively.

$$H = -\sum p_i (\ln p_i) \quad (2)$$

$$E = H/H_{max} = H/\ln R_S \quad (3)$$

where p_i is the ratio of the activity on each substrate (OD_i) to the sum of activities on all of the substrates ($\sum ODi$) and R_S is the number of oxidized C substrates.

Indices adopted from **Orwin and Wardle (2004)** were used to evaluate the resistance (RS) of measured activities to disturbances caused by antibiotic and/or bacterial strain on days 1, 15, 30, 60 and 90, and resilience (RL) indicating recovery of balance, on day 90 after contamination with vancomycin and /or *C. freundii*. The RS index was calculated according to the equation (Eq. 4).

$$RS(t_0) = 1 - 2|D_0|/(C_0 + |D_0|) \quad (4)$$

where D_0 is the difference between the control (C_0) and the disturbed soil (P_0) at the end of the disturbance (t_0). This index of resistance is bounded by −1 and +1, with a value of +1 showing that the disturbance had no effect (maximal resistance), and lower values showing stronger effects (less resistance). If the value of the disturbed soil (P_0) is between 0 and $2C_0$ (i.e. $|D_0|\leq C_0$), the index will give values between 0 and 1. An index value of 0 indicates either a 100% reduction or increase in the value of the disturbed soil. If, however, the value of P_0 is higher than $2C_0$ (i.e. where $|D_0|>C_0$), the index will give a negative value.

The RL index was calculated according to the equation (Eq. 5).

$$RL(t_x) = 2|D_0|/(|D_0| + |D_x|) - 1$$

where D_0 is as above and D_x is the difference between the control (C_x) and the disturbed soil (P_x) at the time point (t_x) chosen to measure resilience. This index is standardized by the amount of change initially caused by the disturbance (D_0), as this determines the state from which it has to recover. This index of resilience is also bounded by −1 and +1. A value of 1 at the time of measurement indicates full recovery (maximal resilience), and lower values indicate a slower rate of recovery. If the absolute value of D_x is between 0 and the absolute value of D_0, the index will give values between 0 and 1. An index value of 0 indicates that the disturbed soil has either not recovered at all since the disturbance ended (i.e. D_0=D_x), or that at t_x it is the same distance away from the control as it was when the disturbance ended at t_0, but in the opposite direction. If the absolute value of D_x is higher than the absolute value of D_0, the index will give a negative value.

Based on the analysis of the kinetics of VA dissipation in soil, its degradation rate was fitted to a zero-order kinetic model. The rate constant (k)/d was determined using the equation (Eq. 6).

$$k = 1/t\ (C_0 - C_t) \quad (6)$$

where C_0 is the amount of VA in the soil at time zero and C_t is the amount of VA in the soil at time t (d). The times at which the VA concentration in soil was reduced by 50% (DT50 values) were calculated from the linear equation obtained from the regression between $C_t - C_0$ of the chemical data and time.

The data were analyzed by applying a three-way analysis of variance (ANOVA) to determine the percentage of the variation that was attributable to the factors being tested (VA concentration, bacterial strain and time). A *post hoc* comparison of the means using the least significant differences (LSD) test was used to assess the statistical significance of differences ($P < 0.05$).

A principal component analysis (PCA) was carried out in two sets using the data for the CLPPs (i.e. AWCD, R_S, H and E), the AWCD data for the six groups in which the 31 carbon substrates of Biolog EcoPlates™ were grouped (i.e. amines, amino acids, carbohydrates, carboxylic acids, miscellaneous and polymers) and the data of enzyme activities from all of the sampling days and was performed separately for each sampling day. Three-way and two-way MANOVA analyses of the PC scores were performed for the first and second PCA sets, respectively. All of the statistical analyses were performed using the Statistica 12.0 PL software package.

REFERENCES

Alef, K. (1995). Dehydrogenase activity. In: Alef K, Nannipieri P (eds) Methods in applied soil microbiology and biochemistry. Academic, London, pp. 228–231

Cycoń, M., Piotrowska-Seget, Z., and Kozdrój, J. (2010). Linuron effects on microbiological characteristics of sandy soils as determined in a pot study. *Ann. Microbiol.* 60, 439–449. doi: 10.1007/s13213-010-0061-0

Cycoń, M., Borymski, S., Orlewska, K., Wasik, T. J., and Piotrowska-Seget, Z. (2016a). An analysis of the effects of vancomycin and/or vancomycin-resistant *Citrobacter freundii* exposure on the microbial community structure in soil. *Front. Microbiol.* 7, 1015. doi: 10.3389/fmicb.2016.01015

Garland, J. L. (1997). Analysis and interpretation of community-level physiological profiles in microbial ecology. *FEMS Microbiol. Ecol.* 24, 289–300. doi: 10.1016/S0168-6496(97)00061-5

Gianfreda, L., Sannino, F., Ortega, N., and Nannipieri, P. (1994). Activity of free and immobilized urease in soil: Effects of pesticides. *Soil Biol. Biochem.* 26, 777–784. doi: 10.1016/0038-0717(94)90273-9

Orwin, K. H., and Wardle, D. A. (2004). New indices for quantifying the resistance and resilience of soil biota to exogenous disturbances. *Soil Biol. Biochem.* 36, 1907–1912. doi: 10.1016/j.soilbio.2004.04.036.

Tabatabai, M. A., and Bremner, J. M. (1969). Use of p-nitrophenyl phosphate for assay of soil phosphatase activity. *Soil Biol. Biochem.* 1, 301–307

Insam, H. (1997). A new set of substrates proposed for community characterization in environmental samples. In: Insam H, Rangger A (eds) Microbial communities: Functional versus structural approaches. Springer-Verlag, Berlin, Germany, pp. 259–260.

Xia, J., Sinelnikov, I. V., Han, B., and Wishart, D. S. (2015). MetaboAnalyst 3.0-making metabolomics more meaningful. *Nucleic Acids Res.* 43, W251–W257. doi: 10.1093/nar/gkv380

TABLE S1. Analysis of variance (three-way ANOVA) for the CLPP indices: average well-color development (AWCD), substrate richness (R_S), Shannon-Wiener (H) and evenness (E) as affected by the bacterial strain (S), concentration (C), time (T) and their interactions.

Parameter	Source of variation	*df*	SS	MS	Variance explained (%)	*F*	*P*
AWCD	S	1	0.03	0.03	6	102.6	0.001***
	C	2	0.07	0.03	17	138.9	0.001***
	T	4	0.06	0.02	17	66.2	0.001***
	S × C	2	0.04	0.02	11	88.4	0.001***
	S × T	4	0.04	0.01	9	36.8	0.001***
	C × T	8	0.04	0.006	11	22.5	0.001***
	S × C × T	8	0.09	0.01	24	47.3	0.001***
R_S	S	1	30.0	30.0	1	1.9	0.176
	C	2	182.9	91.4	7	5.7	0.005**
	T	4	147.2	36.8	6	2.3	0.069
	S × C	2	35.4	17.7	1	1.1	0.338
	S × T	4	265.4	66.4	10	4.1	0.005**
	C × T	8	434.4	54.3	16	3.4	0.003**
	S × C × T	8	595.9	74.5	22	4.7	0.001***
H	S	1	0.4	0.4	2	4.2	0.046*
	C	2	1.1	0.6	7	6.4	0.003**
	T	4	1.7	0.4	10	4.8	0.002**
	S × C	2	0.4	0.2	2	2.1	0.136
	S × T	4	1.6	0.4	10	4.7	0.002**
	C × T	8	2.1	0.3	13	3.1	0.006**
	S × C × T	8	3.8	0.5	23	5.5	0.001***
E	S	1	0.01	0.01	2	3.2	0.081
	C	2	0.03	0.01	4	3.0	0.055
	T	4	0.2	0.04	22	8.2	0.001***
	S × C	2	0.01	0.005	1	1.0	0.383
	S × T	4	0.07	0.02	10	3.8	0.008**
	C × T	8	0.06	0.01	8	1.5	0.166
	S × C × T	8	0.09	0.01	12	2.3	0.0328

Asterisks represent significance level according to ANOVA (**P* < 0.05, ***P* < 0.01 and ****P* < 0.001).

TABLE S2. Analysis of variance (two-way ANOVA) for the RS indices as affected by the treatment (Tr), time (T) and their interactions.

**Parameter**	**Source of variation**	***df***	**SS**	**MS**	**Variance explained (%)**	***F***	***P***
AWCD (overall)	Tr	4	0.30	0.08	5	9.31	0.001***
	T	4	3.82	0.96	63	117.00	0.001***
	Tr × T	16	1.53	0.10	25	11.70	0.001***
Substrate richness (R_S)	Tr	4	2.62	0.66	30	14.14	0.001***
	T	4	1.44	0.36	16	7.77	0.001***
	Tr × T	16	2.42	0.15	27	3.26	0.001***
Shannon-Wiener index (H)	Tr	4	0.77	0.19	22	14.54	0.001***
	T	4	0.76	0.19	22	14.47	0.001***
	Tr × T	16	1.29	0.08	37	6.14	0.001***
Evenness (E)	Tr	4	0.13	0.03	15	4.51	0.003**
	T	4	0.20	0.05	23	6.84	0.001***
	Tr × T	16	0.17	0.01	19	1.42	0.172
AWCD – amines	Tr	4	0.07	0.02	1	0.43	0.783
	T	4	2.52	0.63	34	14.92	0.001***
	Tr × T	16	2.74	0.17	37	4.06	0.001***
AWCD – amino acids	Tr	4	0.88	0.22	6	5.15	0.001**
	T	4	6.02	1.50	44	35.28	0.001***
	Tr × T	16	4.66	0.29	34	6.82	0.001***
AWCD – carbohydrates	Tr	4	0.18	0.05	1	0.35	0.844
	T	4	7.30	1.83	46	13.76	0.001***
	Tr × T	16	1.63	0.10	10	0.77	0.712
AWCD – carboxylic acids	Tr	4	0.87	0.22	6	5.89	0.001***
	T	4	10.55	2.64	74	71.86	0.001***
	Tr × T	16	0.93	0.06	7	1.59	0.1068
AWCD – miscellaneous	Tr	4	0.42	0.10	7	5.96	0.001***
	T	4	4.29	1.07	71	61.38	0.001***
	Tr × T	16	0.49	0.03	8	1.76	0.0653
AWCD – polymers	Tr	4	0.63	0.16	9	6.35	0.001***
	T	4	1.15	0.29	17	11.47	0.001***
	Tr × T	16	3.82	0.24	56	9.57	0.001***
DHA activity	Tr	4	0.35	0.09	28	83.67	0.001***
	T	4	0.60	0.15	49	144.37	0.001***
	Tr × T	16	0.24	0.01	19	14.25	0.001***
PHOS-H activity	Tr	4	0.31	0.08	50	153.05	0.010**
	T	4	0.11	0.03	17	51.37	0.010**
	Tr × T	16	0.19	0.01	29	22.62	0.010**
PHOS-OH activity	Tr	4	0.22	0.05	47	115.43	0.001***
	T	4	0.07	0.02	15	36.51	0.001***
	Tr × T	16	0.16	0.01	34	20.88	0.001***
URE activity	Tr	4	0.98	0.25	54	344.72	0.010**
	T	4	0.43	0.11	24	150.14	0.010**
	Tr × T	16	0.37	0.02	20	32.41	0.010**

Asterisks represent significance level according to ANOVA (**$P < 0.01$ and ***$P < 0.001$).

TABLE S3. Analysis of variance (three-way MANOVA) for the PC1 and PC2 based on the data of CLPP indices for all of the sampling days as affected by the bacterial strain (S), concentration (C), time (T) and their interactions.

Parameter	Source of variation	*df*	SS	MS	Variance explained (%)	*F*	*P*
PC1	S	1	2.9	2.9	1	3.0	0.089
	C	2	18.9	9.5	10	9.9	0.001***
	T	4	23.7	5.9	12	6.2	0.001***
	S × C	2	2.7	1.3	1	1.4	0.252
	S × T	4	17.0	4.2	9	4.4	0.003**
	C × T	8	21.4	2.7	11	2.8	0.010*
	S × C × T	8	46.5	5.8	24	6.1	0.001***
PC2	S	1	4.4	4.4	5	28.1	0.001***
	C	2	11.5	5.8	13	36.4	0.001***
	T	4	17.5	4.4	19	27.7	0.001***
	S × C	2	8.8	4.4	10	27.8	0.001***
	S × T	4	8.9	2.2	10	14.2	0.001***
	C × T	8	9.2	1.1	10	7.3	0.001***
	S × C × T	8	22.0	2.7	24	17.4	0.001***

Asterisks represent significance level according to ANOVA (**$P < 0.01$ and ***$P < 0.001$).

TABLE S4. Analysis of variance (two-way MANOVA) for the PC1 and PC2 based on the data of CLPP indices for each sampling day as affected by the bacterial strain (S), concentration (C), time (T) and their interaction (S × C).

Parameter	Day	Source of variation	*df*	SS	MS	Variance explained (%)	*F*	*P*
PC1	1	S	1	15.0	15.0	43	26.4	0.000***
		C	2	12.7	6.4	37	11.2	0.002**
		S × C	2	0.014	0.007	< 1	0.0	0.987
	15	S	1	0.2	0.2	< 1	0.2	0.637
		C	2	31.9	15.9	72	17.4	0.000***
		S × C	2	1.1	0.6	3	0.6	0.552
	30	S	1	11.2	11.2	32	7.0	0.021*
		C	2	4.4	2.2	13	1.4	0.286
		S × C	2	0.6	0.3	2	0.2	0.830
	60	S	1	1.4	1.4	3	0.8	0.381
		C	2	0.4	0.2	1	0.1	0.885
		S × C	2	23.8	11.9	52	7.1	0.009**
	90	S	1	3.9	3.9	9	6.9	0.022*
		C	2	13.1	6.6	29	11.7	0.002**
		S × C	2	21.2	10.6	47	18.9	0.000***
PC2	1	S	1	1.8	1.8	6	2.2	0.160
		C	2	5.6	2.8	20	3.5	0.065
		S × C	2	10.9	5.4	39	6.8	0.011*
	15	S	1	2.4	2.4	14	5.1	0.043*
		C	2	1.4	0.7	8	1.6	0.250
		S × C	2	7.7	3.8	45	8.4	0.005**
	30	S	1	0.1	0.1	< 1	0.1	0.729
		C	2	10.7	5.3	53	11.1	0.002**
		S × C	2	3.8	1.9	19	3.9	0.050*
	60	S	1	2.5	2.5	13	2.6	0.136
		C	2	3.5	1.8	19	1.8	0.203
		S × C	2	1.3	0.7	7	0.7	0.524
	90	S	1	1.7	1.7	9	4.3	0.060
		C	2	6.0	3.0	31	7.6	0.007**
		S × C	2	6.8	3.4	35	8.6	0.005**

Asterisks represent significance level according to ANOVA (*$P < 0.05$, **$P < 0.01$ and ***$P < 0.001$).

TABLE S5. Analysis of variance (three-way ANOVA) for the carbon substrate groups from the Biolog EcoPlates as affected by the bacterial strain (S), concentration (C), time (T) and their interactions.

Parameter	Source of variation	*df*	SS	MS	Variance explained (%)	*F*	*P*
Amines	S	1	0.01	0.01	2	5.2	0.026*
	C	2	0.03	0.01	4	7.6	0.001**
	T	4	0.16	0.04	23	20.3	0.001***
	S × C	2	0.03	0.02	5	9.0	0.001***
	S × T	4	0.04	0.01	6	5.4	0.001**
	C × T	8	0.11	0.01	17	7.3	0.001***
	S × C × T	8	0.17	0.02	25	10.9	0.001***
Amino acids	S	1	0.02	0.02	4	77.9	0.001***
	C	2	0.13	0.06	20	207.2	0.001***
	T	4	0.12	0.03	19	100.9	0.001***
	S × C	2	0.03	0.01	4	43.8	0.001***
	S × T	4	0.07	0.02	11	55.6	0.001***
	C × T	8	0.15	0.02	24	62.5	0.001***
	S × C × T	8	0.11	0.01	17	43.7	0.001***
Carbohydrates	S	1	0.02	0.02	6	49.0	0.001***
	C	2	0.01	0.01	3	14.6	0.001***
	T	4	0.06	0.01	13	28.4	0.001***
	S × C	2	0.05	0.03	12	51.8	0.001***
	S × T	4	0.07	0.02	18	38.5	0.001***
	C × T	8	0.10	0.01	23	24.9	0.001***
	S × C × T	8	0.08	0.01	19	20.8	0.001***
Carboxylic acid	S	1	0.004	0.004	2	9.1	0.004**
	C	2	0.01	0.004	3	8.0	0.001**
	T	4	0.04	0.01	20	22.5	0.001***
	S × C	2	0.02	0.01	10	23.6	0.001***
	S × T	4	0.03	0.01	13	14.4	0.001***
	C × T	8	0.03	0.00	16	9.1	0.001***
	S × C × T	8	0.05	0.01	23	13.5	0.001***
Miscellaneous	S	1	0.03	0.03	3	9.9	0.003**
	C	2	0.14	0.07	14	26.8	0.001***
	T	4	0.20	0.05	21	19.5	0.001***
	S × C	2	0.10	0.05	11	19.7	0.001***
	S × T	4	0.04	0.01	4	3.8	0.008**
	C × T	8	0.17	0.02	18	8.5	0.001***
	S × C × T	8	0.13	0.02	13	6.2	0.001***
Polymers	S	1	0.09	0.09	8	78.3	0.001***
	C	2	0.28	0.14	24	120.7	0.001***
	T	4	0.06	0.02	5	13.5	0.001***
	S × C	2	0.07	0.03	6	30.1	0.001***
	S × T	4	0.05	0.01	4	10.9	0.001***
	C × T	8	0.04	0.01	4	4.9	0.001***
	S × C × T	8	0.50	0.06	43	54.0	0.001***

Asterisks represent significance level according to ANOVA (*$P < 0.05$, **$P < 0.01$ and ***$P < 0.001$).

TABLE S6. Analysis of variance (three-way MANOVA) for the PC1 and PC2 based on the data of the carbon substrate groups from the Biolog EcoPlates for all of the sampling days as affected by the bacterial strain (S), concentration (C), time (T) and their interactions.

**Parameter**	**Source of variation**	***df***	**SS**	**MS**	**Variance explained (%)**	***F***	***P***
PC1	S	1	18.9	18.9	6	102.6	0.001***
	C	2	49.6	24.8	15	134.8	0.001***
	T	4	59.1	14.8	18	80.3	0.001***
	S × C	2	38.4	19.2	12	104.3	0.001***
	S × T	4	35.8	8.9	11	48.6	0.001***
	C × T	8	44.2	5.5	13	30.1	0.001***
	S × C × T	8	73.7	9.2	22	50.1	0.001***
PC2	S	1	0.2	0.2	< 1	1.5	0.228
	C	2	5.0	2.5	7	18.2	0.001***
	T	4	13.9	3.5	21	25.4	0.001***
	S × C	2	2.0	1.0	3	7.3	0.001**
	S × T	4	3.4	0.9	5	6.3	0.001***
	C × T	8	15.2	1.9	23	13.9	0.001***
	S × C × T	8	18.6	2.3	28	17.1	0.001***

Asterisks represent significance level according to ANOVA (**$P < 0.01$ and ***$P < 0.001$).

TABLE S7. Analysis of variance (two-way MANOVA) for the PC1 and PC2 based on the data of the carbon substrate groups from the Biolog EcoPlates for each sampling day as affected by the bacterial strain (S), concentration (C), time (T) and their interaction (S × C).

**Parameter**	**Day**	**Source of variation**	***df***	**SS**	**MS**	**Variance explained (%)**	***F***	***P***
PC1	1	S	1	17.3	17.3	31	92.9	0.001***
		C	2	24.5	12.2	44	65.6	0.001***
		S × C	2	11.3	5.7	20	30.3	0.001***
	15	S	1	18.7	18.7	22	75.2	0.001***
		C	2	20.3	10.1	23	40.7	0.001***
		S × C	2	44.5	22.2	51	89.2	0.001***
	30	S	1	11.7	11.7	17	120.5	0.001***
		C	2	29.3	14.7	42	151.3	0.001***
		S × C	2	27.1	13.6	39	139.9	0.001***
	60	S	1	1.4	1.4	5	1.2	0.300
		C	2	9.5	4.8	32	4.0	0.047*
		S × C	2	4.4	2.2	15	1.9	0.199
	90	S	1	0.0	0.0	< 1	0.0	0.889
		C	2	17.7	8.8	43	6.8	0.011*
		S × C	2	8.1	4.1	20	3.1	0.080
PC2	1	S	1	1.3	1.3	6	0.9	0.353
		C	2	4.3	2.1	19	1.5	0.254
		S × C	2	0.6	0.3	3	0.2	0.803
	15	S	1	0.0	0.0	< 1	0.0	0.893
		C	2	2.5	1.2	27	3.0	0.088
		S × C	2	1.9	0.9	20	2.3	0.147
	30	S	1	0.5	0.5	2	2.6	0.133
		C	2	9.3	4.7	43	24.2	0.001***
		S × C	2	9.5	4.7	44	24.5	0.001***
	60	S	1	8.8	8.8	42	26.3	0.001***
		C	2	8.0	4.0	38	11.9	0.001**
		S × C	2	0.4	0.2	2	0.5	0.602
	90	S	1	3.1	3.1	10	4.8	0.049*
		C	2	13.0	6.5	42	10.2	0.003**
		S × C	2	7.0	3.5	23	5.4	0.021*

Asterisks represent significance level according to ANOVA (*$P < 0.05$, **$P < 0.01$ and ***$P < 0.001$).

TABLE S8. Analysis of variance (three-way ANOVA) for the enzyme activities as affected by the bacterial strain (S), concentration of vancomycin (C), time (T) and their interactions.

Parameter	Source of variation	*df*	SS	MS	Variance explained (%)	*F*	*P*
DHA	Strain (S)	1	3.4	3.4	< 1	2.2	0.148
	Concentration (C)	2	121.5	60.8	10.8	37.9	0.001***
	Time (T)	4	379.3	94.8	33.7	59.2	0.001***
	S × C	2	36.1	18.1	3.2	11.3	0.001***
	S × T	4	26.5	6.6	2.4	4.	0.005**
	C × T	8	411.0	51.4	36.5	32.1	0.001***
	S × C × T	8	50.9	6.4	4.5	3.9	0.001**
PHOS-H	Strain (S)	1	53	53	< 1	3.3	0.075
	Concentration (C)	2	59	30	< 1	1.9	0.167
	Time (T)	4	7323	1831	57.0	113.9	0.001***
	S × C	2	19	9	< 1	0.6	0.564
	S × T	4	154	38	1.2	2.4	0.061
	C × T	8	4028	503	31.4	31.3	0.001***
	S × C × T	8	239	30	1.9	1.9	0.084
PHOS-OH	Strain (S)	1	43	43	< 1	2.2	0.147
	Concentration (C)	2	955	477	5.9	24.2	0.001***
	Time (T)	4	11352	2838	70.2	143.6	0.001***
	S × C	2	6	3	< 1	0.2	0.859
	S × T	4	60	15	< 1	0.8	0.555
	C × T	8	2456	307	15.2	15.5	0.001***
	S × C × T	8	117	15	< 1	0.7	0.654
URE	Strain (S)	1	0.9	0.9	< 1	0.3	0.568
	Concentration (C)	2	625.5	312.7	27.7	114.4	0.001***
	Time (T)	4	1225.0	306.2	54.2	112.0	0.001***
	S × C	2	3.8	1.9	< 1	0.7	0.503
	S × T	4	17.0	4.3	< 1	1.6	0.197
	C × T	8	209.5	26.2	9.3	9.6	0.001***
	S × C × T	8	13.4	1.7	< 1	0.6	0.763

Asterisks represent significance level according to ANOVA (**P* < 0.05, ***P* < 0.01 and ****P* < 0.001).

TABLE S9. Analysis of variance (three-way MANOVA) for the PC1 and PC2 based on the data of enzyme activities for all of the sampling days as affected by the bacterial strain (S), concentration of vancomycin (C), time (T) and their interactions.

Parameter	Source of variation	*df*	SS	MS	Variance explained (%)	*F*	*P*
PC1	Strain (S)	1	0.9	0.9	0.4	9.8	0.003**
	Concentration (C)	2	5.0	2.5	2.3	27.5	0.001***
	Time (T)	4	151.5	37.9	68.4	416.4	0.001***
	S × C	2	1.3	0.7	0.6	7.1	0.002**
	S × T	4	1.2	0.3	0.6	3.4	0.015*
	C × T	8	52.6	6.6	23.8	72.3	0.001***
	S × C × T	8	3.4	0.4	1.6	4.7	0.001***
PC2	Strain (S)	1	0.1	0.01	0.01	0.1	0.783
	Concentration (C)	2	33.7	16.9	30.8	109.3	0.001***
	Time (T)	4	36.4	9.1	33.2	58.9	0.001***
	S × C	2	0.5	0.2	0.4	1.5	0.233
	S × T	4	2.2	0.5	2.0	3.5	0.0128
	C × T	8	25.8	3.2	23.5	20.9	0.001***
	S × C × T	8	1.9	0.2	1.7	1.5	0.176

Asterisks represent significance level according to ANOVA (**P* < 0.05, ***P* < 0.01 and ****P* < 0.001).

TABLE S10. Analysis of variance (two-way MANOVA) for the PC1 and PC2 based on the data of enzyme activities for each of the sampling days as affected by the bacterial strain (S), concentration of vancomycin (C), time (T) and their interaction (S × C).

Parameter	Day	Source of variation	*df*	SS	MS	Variance explained (%)	*F*	*P*
PC1	1	Strain (S)	1	0.4	0.4	0.8	3.4	0.090
		Concentration (C)	2	52.7	26.4	93.4	210.6	0.001***
		S × C	2	1.8	0.9	3.1	7.1	0.009**
	15	Strain (S)	1	0.008	0.008	0.02	0.03	0.870
		Concentration (C)	2	48.7	24.3	91.3	81.7	0.001***
		S × C	2	1.1	0.5	2.0	1.8	0.209
	30	Strain (S)	1	1.7	1.7	3.5	13.9	0.003**
		Concentration (C)	2	45.0	22.5	92.5	183.5	0.001***
		S × C	2	0.5	0.2	1.0	2.0	0.182
	60	Strain (S)	1	0.3	0.3	0.5	1.5	0.243
		Concentration (C)	2	45.7	22.8	92.6	132.9	0.001***
		S × C	2	1.3	0.7	2.7	3.9	0.050*
	90	Strain (S)	1	0.2	0.2	0.6	0.1	0.785
		Concentration (C)	2	0.6	0.3	2.2	0.1	0.872
		S × C	2	0.1	0.04	0.3	0.02	0.981
PC2	1	Strain (S)	1	1.7	1.7	29.2	8.8	0.012*
		Concentration (C)	2	0.1	0.0	1.0	0.1	0.866
		S × C	2	1.8	0.9	29.9	4.5	0.035*
	15	Strain (S)	1	2.7	2.7	30.4	8.3	0.014*
		Concentration (C)	2	0.6	0.3	6.5	0.9	0.437
		S × C	2	1.7	0.9	19.1	2.6	0.115
	30	Strain (S)	1	4.4	4.4	29.2	7.5	0.018*
		Concentration (C)	2	1.4	0.7	9.1	1.2	0.342
		S × C	2	2.3	1.1	15.1	1.9	0.185
	60	Strain (S)	1	1.8	1.8	17.7	3.5	0.085
		Concentration (C)	2	0.6	0.3	5.3	0.5	0.600
		S × C	2	1.7	0.9	16.8	1.7	0.228
	90	Strain (S)	1	0.7	0.7	3.7	0.5	0.476
		Concentration (C)	2	2.3	1.1	12.4	0.9	0.431
		S × C	2	0.3	0.1	1.5	0.1	0.896

Asterisks represent significance level according to ANOVA (*$P < 0.05$, **$P < 0.01$ and ***$P < 0.001$).

TABLE S11. Recovery level of vancomycin in fortified soil samples.

Nominal concentration (mg/kg)	Replicates/Determined concentration of VA (mg/kg)					Average (mg/kg)	Recovery (%)	SD (mg/kg)	RSD (%)
	1	2	3	4	5				
Control	0.000	0.000	0.000	0.000	0.000	0.000	--	0.000	--
0.1	0.087	0.089	0.094	0.085	0.081	0.087	87.2	0.005	5.7
1.0	0.936	0.961	0.938	0.938	0.956	0.946	94.6	0.012	1.2
10.0	10.512	10.506	10.026	10.519	9.441	10.201	102.0	0.474	4.6

LOQ = 0.1 mg/kg, LOD = 0.01 mg/kg

TABLE S12. Analysis of variance (three-way ANOVA) for the DT50 of the disappearance of vancomycin in soil as affected by the type of soil (TS), bacterial strain (S), concentration (C) and their interactions.

Source of variation	*df*	SS	MS	Variance explained (%)	*F*	*P*
TS	1	3817.8	3817.8	83.1	8103.1	0.000***
S	1	396.6	396.6	8.6	841.7	0.000***
C	1	< 0.1	< 0.1	< 1	0.1	0.801
TS × S	1	372.1	372.1	8.1	789.7	0.000***
TS × C	1	< 0.1	< 0.1	< 1	< 0.1	1.000
S × C	1	< 0.1	< 0.1	< 1	< 0.1	0.774
TS × S × C	1	< 0.1	< 0.1	< 1	< 0.1	0.788

Asterisks represent significance level according to ANOVA (****P* < 0.001).

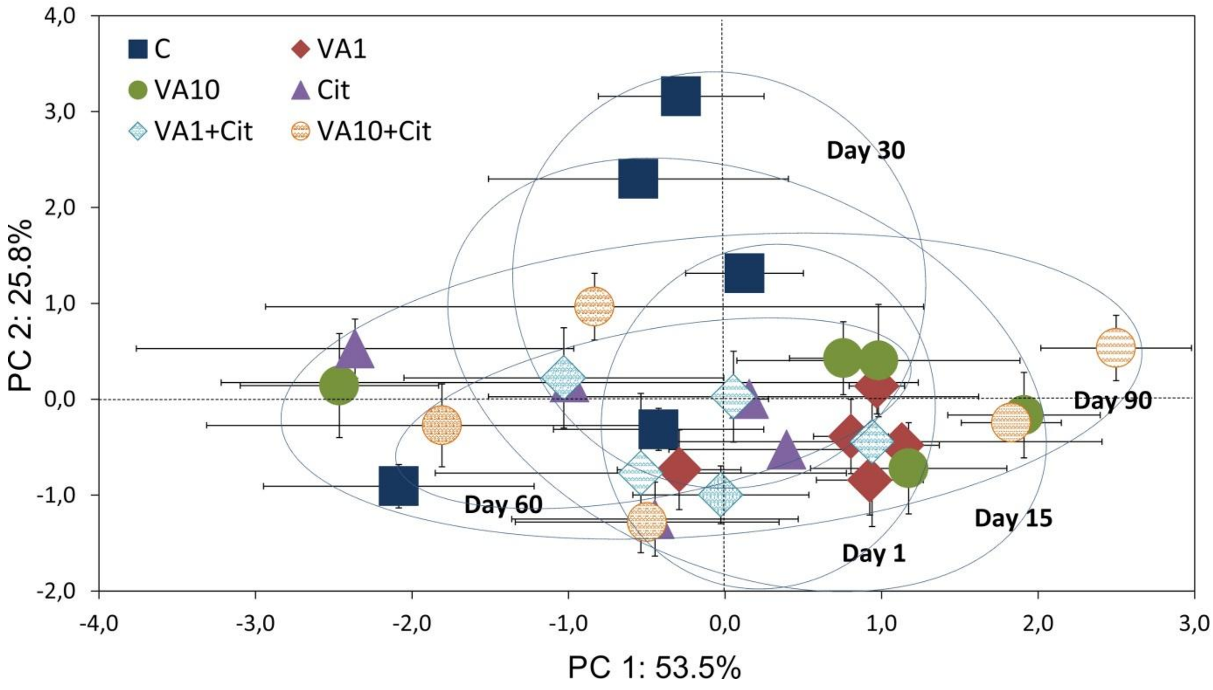

FIGURE S1. Results of the PCA analysis based on the data of the CLPP indices for all of the sampling days. Abbreviations: C – control, VA1 –1 mg VA/kg soil, VA10 –10 mg VA/kg soil, Cit – *C. freundii*, VA1+Cit –1 mg VA/kg soil + *C. freundii*, VA10+Cit –10 mg VA/kg soil) + *C. freundii*. The ovals were used to group treatments from individual sampling days.

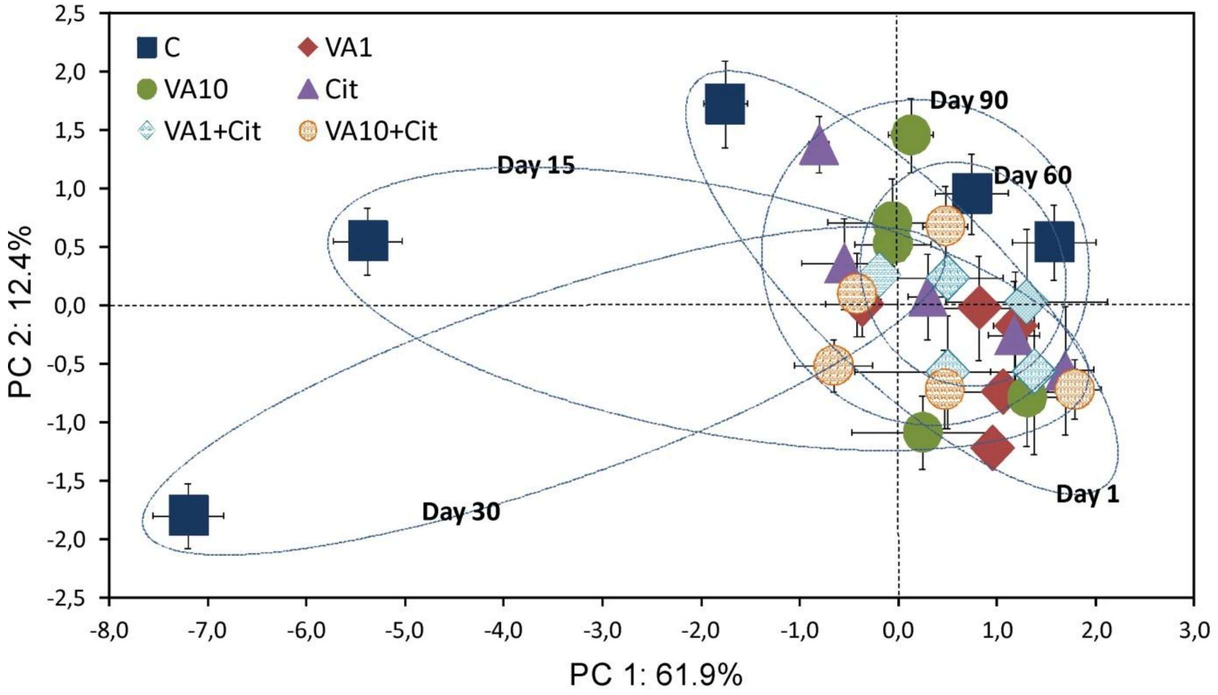

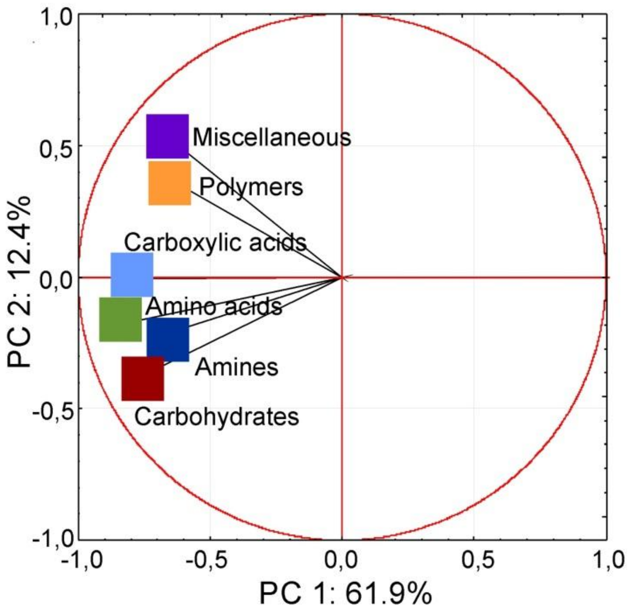

FIGURE S2. Results of the PCA analysis based on the data of the carbon substrate groups for all of the sampling days. Abbreviations: C – control, VA1 –1 mg VA/kg soil, VA10 –10 mg VA/kg soil, Cit – *C. freundii*, VA1+Cit –1 mg VA/kg soil + *C. freundii*, VA10+Cit –10 mg VA/kg soil) + *C. freundii*. The ovals were used to group treatments from individual sampling days.

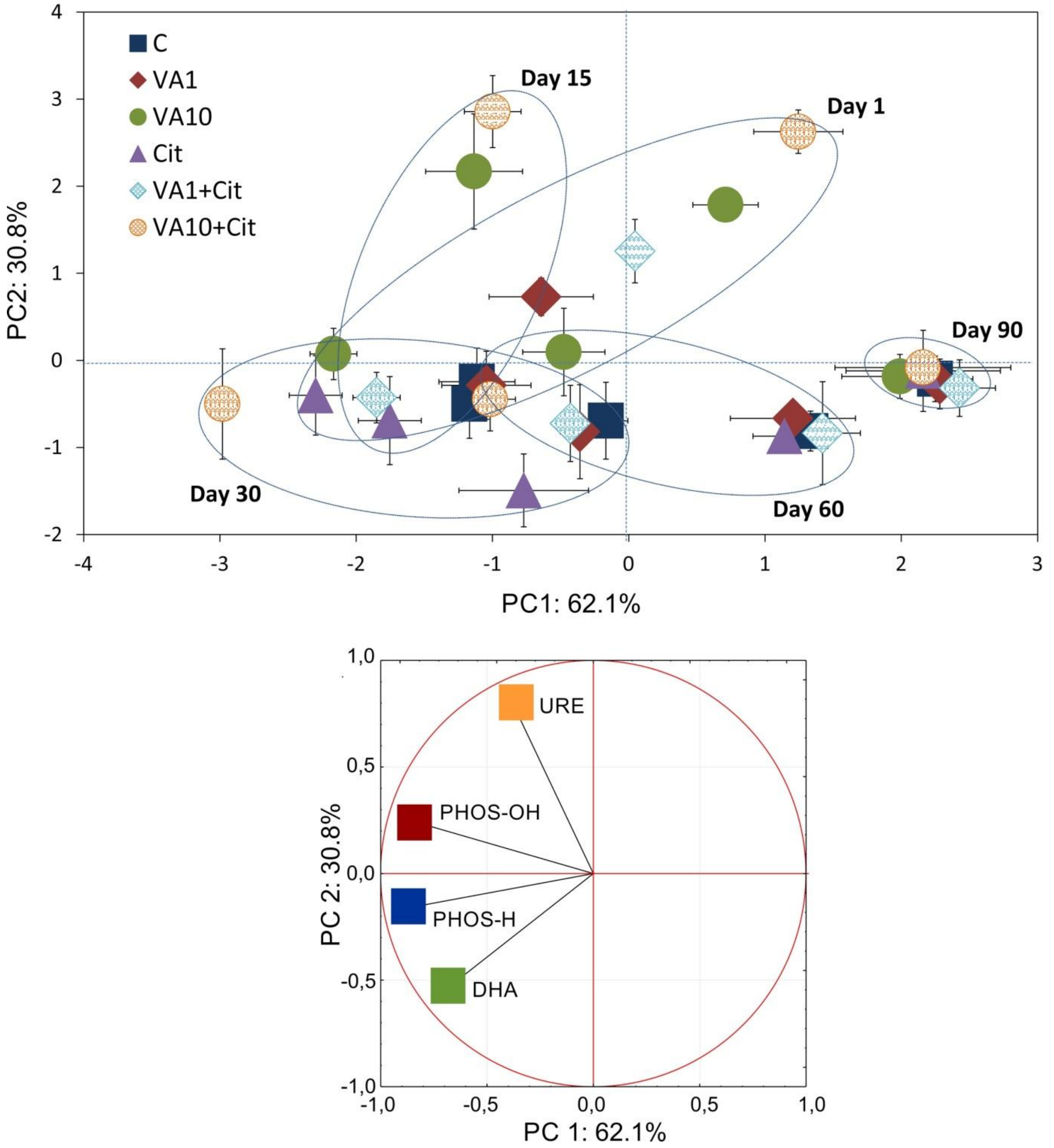

FIGURE S3. Results of the PCA analysis based on the data of the enzyme activities for all of the sampling days. Abbreviations: C – control, VA1 –1 mg VA/kg soil, VA10 –10 mg VA/kg soil, Cit – *C. freundii*, VA1+Cit –1 mg VA/kg soil + *C. freundii*, VA10+Cit –10 mg VA/kg soil) + *C. freundii*. The ovals were used to group treatments from individual sampling days.

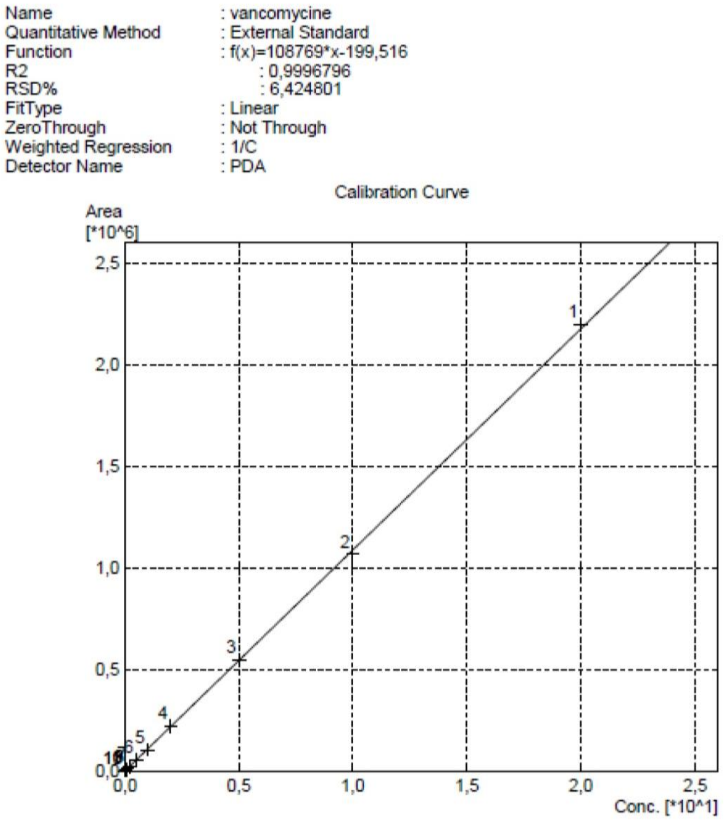

FIGURE S4. Calibration curve for determining the vancomycin that was obtained during the validation studies.

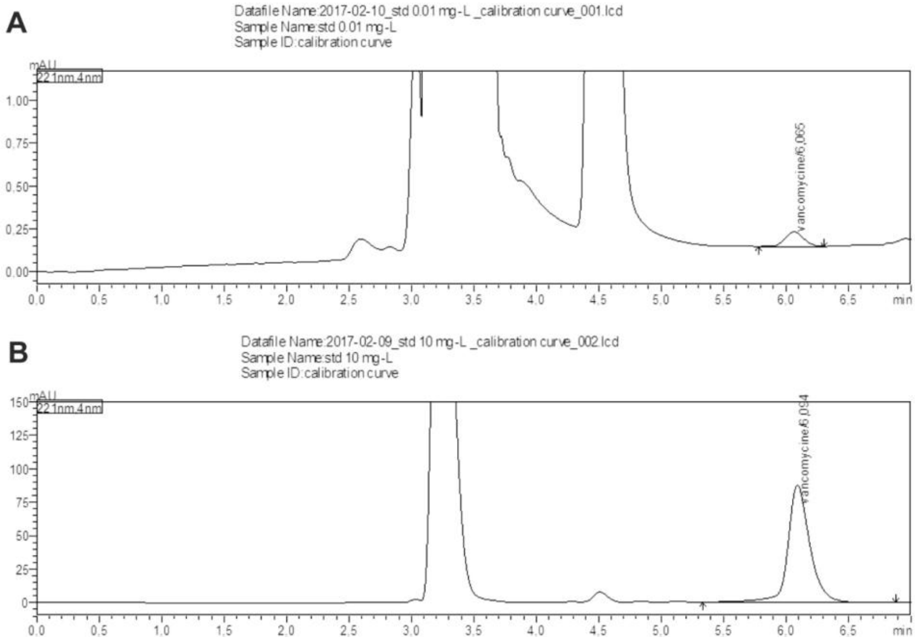

FIGURE S5. Chromatograms for the vancomycin standard at concentrations of 0.01 mg/L (A) and 10 mg/mL (B) that were obtained during the validation studies.

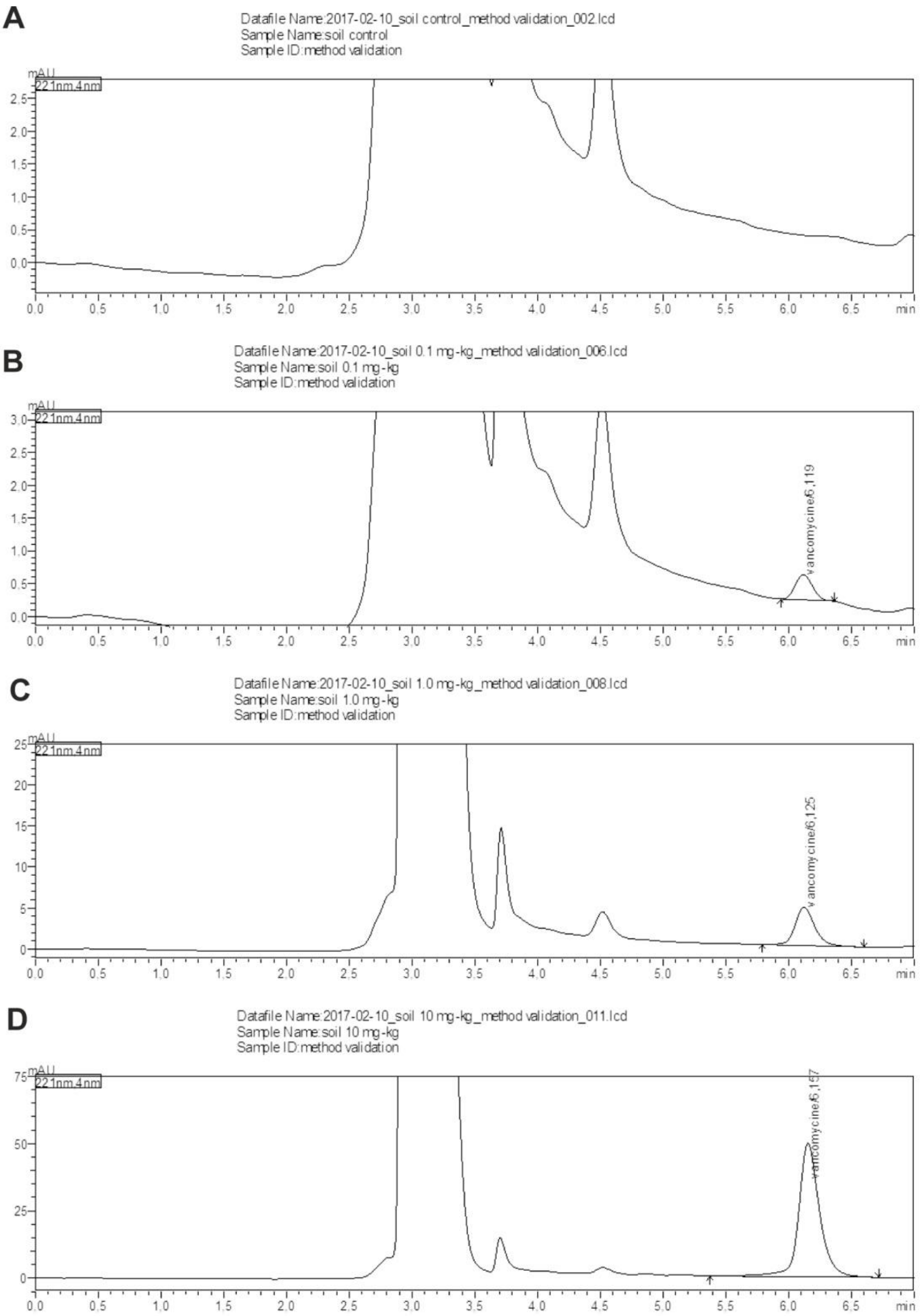

FIGURE S6. Chromatograms for the control (A) and vancomycin-treated soil samples at concentrations of 0.1 mg/kg soil (B), 1 mg/kg soil (C) and 10 mg/kg soil (D) soil that were obtained during the validation studies.
